# Functional Connectivity of the Chemosenses: A Review

**DOI:** 10.3389/fnsys.2022.865929

**Published:** 2022-06-22

**Authors:** Michael C. Farruggia, Robert Pellegrino, Dustin Scheinost

**Affiliations:** ^1^Interdepartmental Neuroscience Program, Yale University, New Haven, CT, United States; ^2^Monell Chemical Senses Center, Philadelphia, PA, United States; ^3^Child Study Center, Yale School of Medicine, New Haven, CT, United States; ^4^Department of Biomedical Engineering, Yale School of Engineering and Applied Science, New Haven, CT, United States; ^5^Department of Radiology & Biomedical Imaging, Yale School of Medicine, New Haven, CT, United States; ^6^Wu Tsai Institute, Yale University, New Haven, CT, United States

**Keywords:** functional connectivity, fMRI, taste, smell, chemesthesis, olfaction, gustation, chemical senses

## Abstract

Functional connectivity approaches have long been used in cognitive neuroscience to establish pathways of communication between and among brain regions. However, the use of these analyses to better understand how the brain processes chemosensory information remains nascent. In this review, we conduct a literature search of all functional connectivity papers of olfaction, gustation, and chemesthesis, with 103 articles discovered in total. These publications largely use approaches of seed-based functional connectivity and psychophysiological interactions, as well as effective connectivity approaches such as Granger Causality, Dynamic Causal Modeling, and Structural Equation Modeling. Regardless of modality, studies largely focus on elucidating neural correlates of stimulus qualities such as identity, pleasantness, and intensity, with task-based paradigms most frequently implemented. We call for further “model free” or data-driven approaches in predictive modeling to craft brain-behavior relationships that are free from *a priori* hypotheses and not solely based on potentially irreproducible literature. Moreover, we note a relative dearth of resting-state literature, which could be used to better understand chemosensory networks with less influence from motion artifacts induced via gustatory or olfactory paradigms. Finally, we note a lack of genomics data, which could clarify individual and heritable differences in chemosensory perception.

## Introduction

The brain can be understood as a network of regions interacting across time and space. These networks can be segregated according to their roles in producing behavior or in precipitating cognition. For example, the default mode network (DMN) has long been found to activate during relaxed, non-task states (Raichle, 2015) and to produce inattentiveness when awry (Metin et al., 2015). Understanding the brain’s functional networks has flourished with the advent of functional magnetic resonance imaging (fMRI) and associated functional connectivity analyses (for a review, see Friston, 2011). Functional connectivity analyses elucidate temporal associations between spatially distinct regions by correlating the time series of their activity (Fingelkurts et al., 2005). These analytic techniques are varied, and include psycho- physiological analysis (PPI), dynamic causal modeling (DCM), and seed-based analyses, among others (Friston et al., 1997, 2003).

Functional connectivity has produced insights in remarkably disparate facets of human behavior, from understanding mechanisms associated with addiction relapse to predicting individual differences in creativity, to better understanding how humans sense the world around them (Sadaghiani et al., 2015; Hsu et al., 2018; Yip et al., 2019). Indeed, within the sensory experience, functional connectivity has illuminated neural processes underlying vision and audition. Such analyses have often described changes associated with pathophysiology, such as in glaucoma (Dai et al., 2013) or hearing loss (Li et al., 2015). Indeed, these fields have been frequently tied together via a singular technique and mode of understanding, yet similar work has been historically infrequent in the chemosenses. Despite this, functional connectivity analyses to better understand how the brain processes taste, smell, and chemesthesis have increased rapidly over the past several years. Since 2000, PubMed’s index of ‘Functional Connectivity’ AND ‘Smell OR Taste OR Chemesthesis’ has increased from 32 results to approximately 1000 (PubMed). We anticipate this trend to increase as chemosensory neuroimaging advances.

In this review, we highlight current work in chemosensory neuroimaging, broken down by sensory system. We pinpoint and summarize the bulk of research being conducted, the techniques used, as well as avenues for future research and exploration.

## Methods

To identify current research in chemosensory neuroimaging, we conducted a review as per PRISMA guidelines (Page et al., 2021, Figure 1). Our full analysis was completed on September 15th, 2021. We conducted our search in six databases, including PubMed, EBSCO, Web of Science, ProQuest, Cochrane, and PsycInfo. Our initial search criteria included the following terms: (human) AND (chemosensory OR olfaction OR gustation) AND (connectome OR connectivity) AND (neuroimaging OR brain OR fMRI OR EEG OR MEG). We implemented these terms in our first searches in PubMed and EBSCO, which concluded on May 20, 2021, and June 20, 2021, respectively. In subsequent searches through Web of Science, ProQuest, Cochrane, and PsycInfo, we broadened our terms to (human) AND (chemosensory OR olfaction OR gustation OR taste OR smell OR odor OR olfact* OR tast* OR gusta*) AND (connectome OR connectivity) AND (neuroimaging OR brain OR fMRI OR EEG OR MEG) to capture additional literature.

**FIGURE 1:**
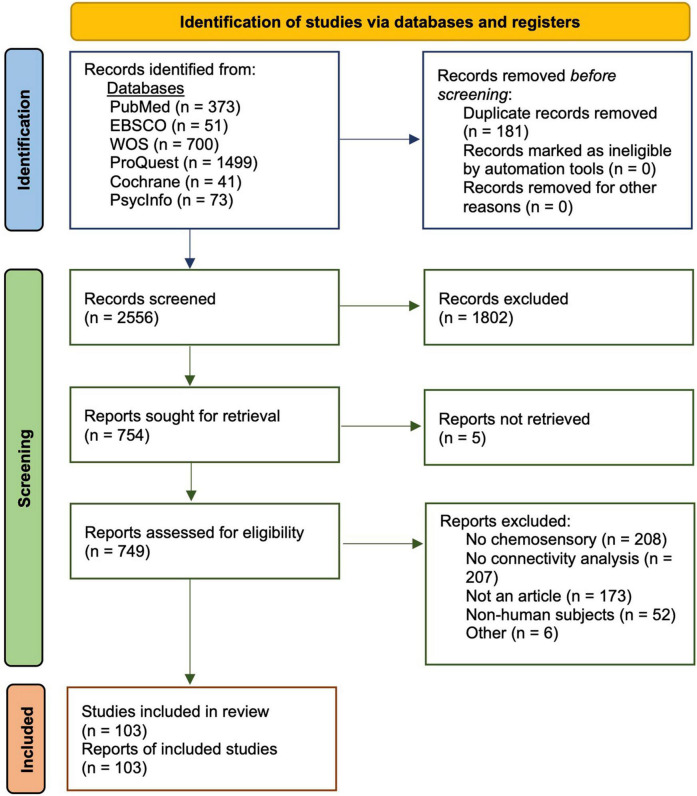
PRISMA flow diagram and relevant search procedures used to obtain 103 chemosensory-related publications with functional connectivity analyses (Page et al., 2021).

In total, we found 373 records from PubMed, 51 from EBSCO, 700 from Web of Science, 8,058 from ProQuest, 41 from Cochrane, and 73 from PsycInfo. Of the 8,058 records from ProQuest, we retained the first 1,499 sorted by relevance and only included peer-reviewed articles from scholarly journals. Following extraction of records from their respective databases, we examined each for relevance by scanning both titles and abstracts. From PubMed, we retained 133 records; from EBSCO, 28; from Cochrane, 21; from PsycInfo, 28; from Web of Science, 110; and finally, from ProQuest, 615. We then removed a total of 181 duplicate titles. In total, 754 records remained to be assessed for eligibility. A total of 749 reports were directly examined following exclusion of unavailable reports. Of these reports, 646 were excluded for many reasons, including lack of chemosensory-related analyses, lack of functional connectivity analyses, review papers/meta-analyses, structural connectivity analyses only, book chapters or abstracts, and non-human animal studies, among others. A total of 103 studies were eligible for inclusion in this review (see Supplementary Table 1 for a full list of studies).

Eligible studies were broken down by sensory systems (olfaction, gustation, chemesthesis, flavor, or a combination of these modalities). Demographics were tallied per system of interest, and additional data categories were assessed, including behavioral, physiological, and genomics data. Imaging data were divided into three categories, task-based, resting-state, or other, and specific connectivity analyses were recorded. Lastly, the accessibility of these data types was noted.

## Results

Of the 103 studies, 48 examined olfaction, 15 gustation, 12 chemesthesis, and 8 flavor. Thus, 20 remaining studies examined a combination of sensory systems (i.e., were multisensory). These 103 studies comprised a total of 4,497 subjects. Mean age and breakdowns by sex were not included for all studies; however, of the 98 studies that included sex breakdowns, a total of 1,944 subjects were male (∼46%) and 2,273 were female (∼54%). From the 94 studies that included mean participant age, we calculated the grand mean age, which was 37.12 years. For summary participant characteristics, see Table 1. In the following paragraphs, we discuss key findings from olfactory, gustatory, chemesthetic, flavor, and multisensory studies, respectively. We furthermore break down each sensory modality into main topics.

**TABLE 1:** Summary of participant and study characteristics.

Modality	Demographics					^8^Connectivity Imaging			Other Data			

	***Number of studies***	***N***	***Sex (% Female)***	***Mean Age***	***Patient***	***Task***	***Resting***	***Other (e.g., EEG)***	***Behavioral***	***Physiological***	***Genomic***	***Open Data***
Olfaction	48	57.15 ± 106.07	^1^54.41%	^2^42.54	43.75%	56.17%	41.67%	14.58%	85.42%	27.08%	0%	16.67%
Gustation	15	27.53 ± 27.79	76.57%	^3^22.75	13%	93.33%	6.67%	0%	73.33%	6.67%	0%	6.67%
Chemesthesis	12	37.17 ± 33.02	46.41%	^4^26.30	25%	83.33%	16.67%	0%	75%	33.33%	16.67%	16.67%
Flavor	8	39.13 ± 24.27	44.41%	23.01	12.50%	87.50%	25%	0%	75%	25%	12.50%	12.50%
Multisensory	20	29.10 ± 14.17	46.56%	^5^33.06	15%	90%	10%	10%	85%	35%	0%	5%
Total	103	43.66 ± 75.22	^6^53.90%	^7^37.12	29.13%	72.82%	26.12%	8074%	81.55%	26.21%	2.91%	12.62%

*^1^43 studies reported data;*

*^2^45 studies reported data;*

*^3^13 studies reported data;*

*^4^9 studies reported data;*

*^5^19 studies reported data;*

*^6^98 studies reported data;*

*^7^94 studies reported data;*

*^8^percentages do not necessarily add to 100, due to studies combining resting-state, task data, or other.*

### Olfaction

Of the 48 studies to examine the olfactory sense, 21 studies were task-based, 16 studies were resting-state, four studies applied connectivity analysis to both imaging data types, and seven used EEG, MEG, or PET. Most resting-state studies (*n* = 12), focused on an unhealthy olfactory system. Similarly, many task-based connectivity studies examined clinical populations (*n* = 6). Studies are broken down into themes of olfactory pleasantness (*n* = 2), anatomy (*n* = 7), emotion (*n* = 4), attention (*n* = 4), pathology (*n* = 18), and other (*n* = 13), respectively. We also mention publications containing open data. For full study characteristics and breakdowns, see Supplementary Table 2.

#### Pleasantness

Among publications touching on olfactory pleasantness is an experimental approach by Carlson et al. (2020), who scanned subjects with pleasant, unpleasant, or no odor across 2 days. The task-based portion was followed by a resting-state scan to examine lingering effects of stimulus valence. A general linear model (or GLM) contrasting odor and non-odor tasks was used to isolate regions of interest (ROIs) for subsequent resting-state connectivity analysis of study conditions: post-no odor, post-unpleasant, and post-pleasant (Carlson et al., 2020). A binary Random Forest model using correlation matrices was implemented to predict positive or negative odor rest-periods vs. no-odor periods. Faster (∼0.031–0.063 Hz) and slower (∼0.016–0.031 Hz) sub-bands were reported to show different network associations among conditions. The precuneus was involved in networks for both bands of the unpleasant odor condition, and more networks were engaged in the unpleasant > pleasant matrix comparison in only the high frequency band. However, the predictive model (area under the curve, AUC = 0.91) revealed that connectivity between left insula and left dorsolateral prefrontal cortex (dlPFC), left amygdala, left parahippocampus, and right hippocampus distinctively increased after subjects experienced unpleasant odors (Carlson et al., 2020).

#### Anatomy

The olfactory bulb is the first relay from olfactory receptor neurons into the brain, and is important for the completion of several olfactory tasks, such as odor discrimination, concentration-invariant recognition, segmentation, and pattern recognition, prior to cortical processing (Wilson and Sullivan, 2011). Using publicly available Human Connectome Project (HCP) data (Van Essen et al., 2013), Weiss et al. (2020) found that a small percentage of left-handed females (4.25%) did not have olfactory bulbs that were visible among structural scans. Using an independent small sample (2 individuals without bulbs) they showed similarity in functional responses to odors to a larger control group; however, due to their small sample size, the connectivity analysis was inconclusive (Weiss et al., 2020). Findings may represent an alternative pathway for olfactory processing prior to cortical information relay, or alternatively, technical issues of low spatial resolution in the processed structural images. A larger sample to explore connectivity similarities may clarify their findings. Additionally, new, non-invasive recording techniques of the human olfactory bulb, coined Electrobulbogram (or EBG) (Iravani et al., 2020), with the appropriate connectivity analysis, may allow researchers to directly test this alternative pathway hypothesis.

Arnold et al. (2020) also examined the HCP dataset to parcellate human olfactory networks across frontal and temporal regions. This was accomplished by correlating 812 individual behavioral olfactory measures (i.e., odor identification) with later accessed resting-state scans. Additionally, by applying graph theory, they illuminated an optimized network with sensory, limbic, and frontal subnetworks (Arnold et al., 2020).

#### Emotion

In two studies, Krusemark and Li (2012) and Krusemark et al. (2013) show that anxiety increases connectivity between piriform cortex and affective brain regions, including amygdala and hippocampus, during odor perception. In the first study, individuals with higher self-reported state anxiety were better at discriminating four negative and two neutral odors. Here, PPI analyses were used. In brief, PPI is a technique that assesses changes in the correlation between a seed region and other brain regions due to a given task or psychological manipulation; if there is a stronger correlation between brain regions during such an event, it suggests an exchange of information (Friston et al., 1997; Lordier et al., 2019). The aforementioned PPI analysis found that anxiety was associated with increased connectivity between posterior piriform cortex (PPC) and amygdala for negative vs. neutral odors (Krusemark and Li, 2012). By inducing anxiety prior to scanning odor responses, a second study replicated the prior study, demonstrating that anxiety creates strong connective engagement of the amygdala (afferent or efferent) with the olfactory system (Krusemark et al., 2013). Similarly, a reduction in insula response (along with emotional response) and its connectivity with associated networks to a disgusting odor may help individuals maintain homeostasis (Meier et al., 2015). These studies imply that connectivity may constitute an olfactory etiology model of emotional disorders.

#### Attention

Several functional connectivity studies of olfaction elucidate the interaction of this sense with the brain’s attention networks. For example, evidence suggests that connectivity between ventrolateral prefrontal cortex (vlPFC) and dorsomedial PFC (dmPFC)/anterior cingulate cortex (ACC) may mitigate the effects of olfactory distractors (Weigard et al., 2021). Such aversive sensory distraction is abated by engaging higher order neural processes that are especially potent under conditions of greater working memory load (Weigard et al., 2021). In a different vein, olfaction and attention interact frequently when subjects are exposed to complex odor mixtures with varied qualities or affective components. For example, exposure to blended essential oils increases fronto-parietal functional connectivity as measured via EEG (Liu et al., 2019). Similarly, attention to a hedonically complex odor mixture (i.e., with both pleasant and unpleasant components) vs. a singularly valenced odor (i.e., pleasant only) was found to modulate functional connectivity between superior frontal gyrus (SFG) and medial orbitofrontal cortex (OFC), bilateral LPFC, and mediodorsal (MD) thalamus (Grabenhorst et al., 2011). Findings from Plailly et al. (2008) similarly bolster evidence suggesting that OFC and MD thalamus play a large role in olfactory attention. Indeed, attention to odor over an auditory stimulus enhances effective connectivity from MD thalamus to OFC in a directionally specific way; further, connectivity between these two regions is weakened during attention to the auditory stimulus relative to the odor (Plailly et al., 2008).

#### Pathology

Olfactory dysfunction in humans is not uncommon, affecting between 5 and 20% of the population depending on severity (Han et al., 2019). Indeed, there have been several MRI investigations of associated structural and functional alterations, with most connectivity studies on olfaction focusing on impairment in neural networks among patients vs. healthy controls (Han et al., 2019). Nearly half of the resting-state data described here has focused on neurodegenerative diseases commonly associated with a decrease in olfactory acuity, including Dementia, Alzheimer’s disease (AD), and Parkinson’s disease (PD) (Supplementary Table 2). This method has proven successful at building neural biomarkers of disease (Woo et al., 2017). Consequently, no task-based designs have measured connectivity among these patients; however, task-based designs of connectivity might have more power in discriminating between groups and predicting individual differences (Greene et al., 2018; Rasero et al., 2018). Resting-state studies show different functional connectivity across neurological disorders as well as differences within them. However, there is little agreement on which networks increase or decrease with olfactory function due to differences in connectivity seeds chosen, patient groups studied, and behavioral tests of olfaction. For instance, a posterior cingulate cortex (PCC) seed showed both positive and negative association between connectivity with other regions and olfactory function in PD patients with impaired smell vs. those with no impairment (Sunwoo et al., 2015). Meanwhile, PD patients with hyposmia had their smell function positively correlated with connectivity of the OFC and insula in comparison to healthy controls when seeds were set on piriform and OFC (Lee et al., 2020). Su et al. (2015) took another approach, defining the seeds as ROIs from *t*-tests between populations of interest, including those with PD and olfactory dysfunction and those with PD but without olfactory dysfunction. They obtained eight ROIs from their analysis, including OFC, PCC, parahippocampal gyrus, left rectal gyrus, superior temporal pole (STP), right insula, amygdala, and inferior frontal gyrus (IFG). From these seeds a threshold measurement of olfaction was determined. This analysis procedure found decreased functional connectivity within limbic and paralimbic cortices (Su et al., 2015).

Other patient populations under study include etiologies of smell loss (e.g., post-viral, post-traumatic) as well as nutrition (e.g., adiposity). General loss of smell, unrelated to neurodegenerative diseases or age, has shown widespread connectivity from piriform cortex to bilateral prefrontal areas, left IFG, and left premotor cortex in response to sniffs, which decreases following olfactory training, a form of smell loss therapy (Kollndorfer et al., 2014). Indeed, olfactory training induces widespread effects on connectivity. Kollndorfer et al. (2015) found an increase in the number of functional connections to somatosensory, olfactory, and integrative networks (i.e., regions responsible for multisensory integration) following olfactory training, mimicking those of healthy controls at baseline. Widespread networks, outside of the piriform cortex, have also been present in other task-based studies that use odor stimuli (Reichert et al., 2018; Park et al., 2019; Pellegrino et al., 2021). Reichert et al. (2018) used independent component analysis (ICA) to isolate three networks active during odor stimulation. In brief, ICA is a technique that is implemented to separate fMRI data into spatially independent components, which allows for the discovery of hidden features or signals (McKeown et al., 1998; Calhoun and Adali, 2006). This analysis yielded sensory processing (including insula, thalamus, and piriform cortex, among others), cerebellar, and occipital networks. These studies contain a heterogeneous patient population including a range of etiologies. Looking at patients with traumatic olfactory loss, Pellegrino et al. (2021) showed that neither piriform activation nor its connectivity predicted loss. However, using Connectome-based Predicting Modeling (CPM), a network outside of the primary olfactory cortex could discriminate between healthy and anosmic patients (with 64% accuracy) (Finn et al., 2015; Shen et al., 2017). Similarly, using resting-state data, Park et al. (2019) showed that functional connectivity in traumatic olfactory loss was increased in sensory and thalamic networks in comparison to healthy controls. Additionally, those patients with worse olfaction showed an increase in global efficiency (i.e., a functional integration metric; Rubinov and Sporns, 2010) and a decrease in modularity (i.e., a functional segregation metric; Blondel et al., 2008).

#### Other

The last several studies within the olfactory domain cover a disparate array of topics, from sleep/consciousness and memory to olfactory expertise and even obesity pathophysiology. We briefly discuss a select few of these studies. Among sleep/consciousness research, odors advertised to promote sleep were found to negatively modulate connectivity from piriform cortex to regions of DMN, such as PCC (Watanabe et al., 2018). Sleep and olfaction can furthermore interact with the endocannabinoid system to promote aberrant food choice. Indeed, connectivity between insula and piriform cortex following odor exposure inversely mediated the relationship between endocannabinoid levels following sleep deprivation and energy density of subsequently consumed foods (Bhutani et al., 2019). Among the senses, memories triggered by olfactory stimuli are among the most salient; using graph-theoretic approaches Meunier et al. (2014) found that successful odor recognition memory was correlated with connectivity among a network comprising ACC, caudate, and hippocampus. Finally, among publications considering olfactory expertise, Min (2003) found that, relative to general workers, professional olfactory researchers displayed enhanced cortico-cortical connectivity (from OFC in particular) to odors. Indeed, while these publications cover differing topics and themes, each are unique and add to the rich literature of functional connectivity in olfaction.

#### Open Data in Olfaction

Of all 48 studies on olfaction, four studies provide new, open-source data while four others based their analysis on open-source data from other groups. One study collected EEG data. Of the remaining studies, all conducted connectivity analysis on resting-state data; however, a study by Carlson et al. (2020) also collected task-based data and made it available for additional analysis^1^.

### Gustation

Of 15 studies examining the gustatory sense, a total of 14 included task-based fMRI while one included resting-state data. Among the 14 task-based studies, a total of two include patient populations. Gustatory studies cover subtopics of taste pleasantness (*n* = 3), gustatory anatomy (*n* = 2), sweeteners (*n* = 2), taste intensity (*n* = 2), attention to taste (*n* = 4), and pathology (*n* = 2); open data is also discussed.

#### Pleasantness

Of the task-based fMRI studies that exclude patients, three use PPI analyses, three use DCM, and six use a combination of PPI, DCM, or other techniques. Among PPI studies, Jabbi et al. (2008) sought to discern neural correlates of emotions through observing (i.e., viewing someone consume a bad tasting stimulus), imagining (non-gustatory), and experiencing (via quinine). Jabbi et al. (2008) took the frontal operculum/anterior insula (FO/AI) as a seed, and used it across modalities to compare connectivity during disgust vs. neutral conditions. For disgusting vs. neutral *taste*, FO/AI was more strongly connected to regions including temporal pole, cingulate (motor), cerebellum, OFC, caudate, putamen, globus pallidus, and posterior insula, among others. Indeed, functional regions differed among observing, imagining, and experiencing; though involvement of FO/AI was ubiquitous in each. Jabbi et al. (2008) conclude that FO/AI is a “convergence zone” for disgust, regardless of whether the stimulus is an aversive taste or the imagination of an aversive experience.

In a 2008 study, Grabenhorst and Rolls found that attention to taste pleasantness vs. intensity is associated with activity in the OFC and pregenual cingulate cortex (PGC) (Grabenhorst and Rolls, 2008). In contrast, attention to intensity vs. pleasantness is correlated with activity in both right anterior and mid insular regions. Grabenhorst and Rolls (2010) then sought to examine the neural origin of higher order attentional biases to taste pleasantness vs. intensity using a monosodium glutamate (MSG) stimulus. PPIs were used, with the aforementioned regions as seeds, to determine additional brain regions that modulate connectivity depending on attentional condition. Attention to pleasantness over intensity was associated with greater correlation between OFC and anterior lateral prefrontal cortex (aLPFC); in a different region of aLPFC, pleasantness over intensity was associated with greater connectivity to PGC (Grabenhorst and Rolls, 2010). In contrast, attention to intensity over pleasantness correlated with greater posterior lateral prefrontal cortex (pLPFC) to AI connectivity. These findings suggest that LPFC is critical in orienting selective attention to the intensity or affective value of a taste stimulus.

Bender et al. (2009) similarly examined brain response to taste during several tasks. However, rather than evaluate pleasantness vs. intensity, subjects either rated stimulus pleasantness, passively received the stimulus, indicated the presence of a stimulus, or noted stimulus identity. Stimuli consisted of either sweet, sour, salty, or tasteless (i.e., artificial saliva) solutions. In brief, a main effect of stimulus was found in AI, and a main effect of task – driven by pleasantness evaluation – was found in lateral OFC (Bender et al., 2009). A PPI analysis was implemented to determine whether AI or lateral OFC influenced other brain regions due to stimulus or task. Stronger bilateral connectivity between left dorsal AI and amygdala was found for passive taste reception vs. active evaluation (Bender et al., 2009). Finally, reception of taste over tasteless solution was associated with greater connectivity between lateral OFC and AI, subcallosal cingulate, and bilateral caudomedial OFC, as well as left ventral striatum (VS). Indeed, these findings underscore the importance of extra-insular regions in central taste processing.

#### Anatomy

Outside of PPIs, three studies used DCM to better understand central gustatory processing. In brief, DCM is an effective connectivity technique that relies on a Bayesian framework to elucidate the architecture of dynamic brain networks (Marreiros et al., 2010). DCM is a model comparison procedure; models are specified based on hypothesized interactions between regions, self-interactions, as well as the potential external influence of psychological variables (Penny et al., 2004). An optimal model of effective connectivity is that which, when convolved with a model of neural dynamics and a hemodynamic “forward model,” minimizes the difference between observed and predicted fMRI time series (Penny et al., 2004; Stephan et al., 2007). First in the series of DCM publications, Nakamura et al. (2013) sought to identify the precise site of primary taste cortex in human insula. To accomplish this, a unique experimental design was developed to maximize signal-to-noise. Both passive and active taste conditions were used during fMRI; subjects passively tasted an umami solution, and were separately asked to identify a salty solution, which did not exist, in an umami solution (Nakamura et al., 2013). Three volumes of interest (VOIs) were identified, including right middle insula (MI), right AI (i.e., activated by taste), and right middle frontal gyrus (MFG)/frontal eye field (FEF) (i.e., activated during attentional tasks). A total of six DCMs were constructed based on these VOIs, with three having taste as an input into MI and three with taste entering AI. All six DCMs had bidirectional intrinsic connections between each VOI. Two DCMs had the taste identification task as modulating connections from MI to AI, two had these on connections from AI to MFG/FEF, and another two had these on both MI to AI and AI to MFG/FEF. Random-effects Bayesian Model Selection (BMS) found a “winning” model with an exceedance probability of 0.793 consisting of taste information entering MI and task information modulating connectivity from both MI to AI and AI to MFG/FEF (Nakamura et al., 2013). In sum, these results provide strong evidence of MI as the locus of primary taste cortex.

Regarding taste quality, Iannilli et al. (2012) examined laterality within the gustatory system. The fMRI paradigm included presentation of supra-threshold umami and salty solutions at left and right sides of the mouth separately (Iannilli et al., 2012). VOIs included left ventroposterior medial nucleus of thalamus (VPM), left FO, and left LPFC, which were derived from a group analysis of the salty solution. Based on fixed and random effects BMS models, the highest posterior probability existed for a model with driving taste input into thalamus, followed by unidirectional intrinsic connections from thalamus to FO, and from FO to LPFC. These findings support ipsilateral processing as previously suggested in group analysis.

#### Sweeteners

Van Opstal et al. (2019) investigate brain response to nutritive vs. non-(or low)nutritive sweeteners to examine neural mechanisms of energy homeostasis. Resting-state fMRI was collected on subjects pre and post consumption of shakes with sweeteners of the aforementioned energy density. Functional networks were compared pre vs. post ingestion. A significant increase in activity of the salience network following consumption of glucose was observed, with no other significant differences. Such changes were observed only following glucose and not sucralose consumption. Taken together, these findings indicate that nutritive sweeteners impact brain connectivity more than their non-nutritive counterparts. While this study did not seek to examine effects of taste quality *per se*, the methods used could yield broad insights for studies of gustation. Frank et al. (2008) also assessed similarities in neural correlates between nutritive and non-nutritive sweeteners. Subjects tasted sucrose or sucralose solutions under fMRI. Seed regions for subsequent functional connectivity analyses were selected from FO/AI, using voxels that were active during sucrose and sucralose consumption. Regarding both sweeteners, there were similar patterns of functional connectivity with FO/AI. Left FO/AI exhibited significant functional connectivity with right insula, left striatum, bilateral ACC, and left thalamus. Conversely, right FO/AI demonstrated significant connectivity with bilateral ACC, thalamus, and contralateral insula. Regarding differences in connectivity between sweeteners, sucralose, compared to sucrose, demonstrated greater functional connectivity from bilateral FO/AI to bilateral anterior VS (Frank et al., 2008).

#### Taste Intensity

Central gustatory relays are responsible for processing additional properties of taste aside from quality; taste *intensity* is one such example. To better understand how insula and thalamus communicate information regarding taste intensity, Yeung et al. (2016) used DCM on fMRI data obtained while participants consumed various salt solutions. DCMs were created based on interactions between three VOIs: insula, thalamus, and postcentral gyrus (PCG); PCG was included due to its consistent activation following consumption of salty solutions (Yeung et al., 2016). The “winning” family model consisted of driving taste inputs into thalamus, bidirectional intrinsic connections from thalamus to insula, and unidirectional intrinsic connections from thalamus and insula to PCG. Further, there was a modulatory impact of taste intensity on connections from insula to insula, and on unidirectional connections from insula to PCG and to thalamus. Indeed, these results are significant as they suggest an effect of taste intensity on connections from insula to thalamus.

Within individuals, intensity ratings for olfactory and gustatory stimuli tend to be highly correlated, yet there exist differences *between* individuals (Green et al., 2005; Veldhuizen et al., 2020). Veldhuizen et al. (2020) argue that, because taste and smell rely on different peripheral receptors and cranial nerves, there must exist a common “central gain mechanism” (CGM) that modulates individual perceptual differences. A primary suspect for this role is amygdala, as resections of amygdala in patients with epilepsy result in *enhanced* intensity perception, suggesting that amygdala tonically inhibits gustatory circuitry (Small et al., 2003). To test this, both PPI and DCM methods were used on fMRI data acquired from subjects who rated intensities of sweet, sour, and salty stimuli (Veldhuizen et al., 2020). PPIs were constructed using seeds from areas exhibiting a main effect of taste minus tasteless, as well as a region of amygdala responsive to taste intensity. Taste intensity ratings were then regressed against connectivity among these regions to determine a functional network responsive to taste intensity. This network consisted of amygdala, MD thalamus, and VPM thalamus/pulvinar. DCMs were then specified to elucidate direction of information flow and influence, with the aforementioned regions as VOIs. The DCM that emerged accounted for 13.7% of the variance in intensity ratings. It consisted of negative connections from left amygdala to bilateral VPM/pulvinar and bilateral MD thalamus. Further, the DCM contained negative connections from left MD thalamus to right VPM/pulvinar and positive bilateral connections from right VPM/pulvinar to right MD thalamus. In total, these results support the existence of a CGM within the amygdala and thalamus that modulates individual differences in taste intensity perception; though, the work by Veldhuizen et al. (2020) did not find evidence for a role of PCG or insula as in Yeung et al. (2016).

#### Attention

Veldhuizen et al. (2011) attempt to uncover the neural locus whereby breaches of taste identity expectation are encoded. To accomplish this, subjects were presented with sweet and tasteless solutions in fMRI, with matched and mismatched stimuli. During breaches of taste identity expectation (i.e., unexpected > expected), *deactivation* of fusiform was observed. In contrast, brain regions that activated more in the unexpected vs. expected contrast included gustatory regions of VPM thalamus and AI, reward-related regions, including OFC and VS, as well as attention-related regions, including IFG, ACC, anterior dorsal insula, and intraparietal sulcus (IPS). From this, Veldhuizen et al. (2011) hypothesized that attention and reward regions modulate sensory regions to promote goal-directed behavior and enhance processing of salient biological stimuli, respectively (Veldhuizen et al., 2011). To test this explicitly, expectancy-dependent changes in connectivity were examined using PPI, with the aforementioned regions as ROIs. When expectations are breached (i.e., unexpected > expected), greater connectivity was observed between right VS and bilateral AI, as well as between left AI and both left VS and right IPS (Veldhuizen et al., 2011). DCM was used to determine how reward and attention-related regions modulate sensory systems, specifically regarding the directionality of information processing. Again, fixed effects BMS was implemented, which found the largest posterior probability of 0.954 for a model consisting of driving input from taste and tasteless into AI, unidirectional input of IPS into AI, and bidirectional intrinsic connections between AI and VS.

Veldhuizen et al. (2012) subsequently sought to better understand how attention and gustatory networks interact to orient individuals to relevant sensory signals. In brief, fMRI data were acquired while subjects were either actively (i.e., noting the presence of a taste via button press) or passively tasting sweet, sour, salty, or tasteless stimuli. PPIs were constructed using seeds from the active vs. passive contrast for tasteless; these regions included bilateral AI, bilateral FO, right FEF, left MFG, left parietal operculum (PO), left posterior parietal cortex (PPC), ACC, and PCC. PPIs demonstrated greater connectivity under active vs. passive tasting conditions between right FEF and ACC, between ACC and right AI, between ACC and right FO, and finally between left PPC and ACC. DCMs were then constructed to determine the direction of information flow through attention and gustatory networks. DCMs emphasized the potential role of the ACC in mediating attention-gustatory interactions due to its prominence in the aforementioned analysis. BMS found a model (i.e., posterior probability ∼1), consisting of driving input from taste and tasteless into AI and bidirectional intrinsic connectivity between FEF and ACC, PPC and ACC, PO and ACC, and AI and ACC (Veldhuizen et al., 2012). These findings suggest that brain regions responsible for attention (i.e., PPC and FEF) exert a top-down influence on gustatory circuitry (i.e., AI) indirectly via an ACC relay.

Granger causality (GC) is a tertiary metric that, like PPI and DCM, has been frequently used to gauge effective connectivity. Conceptually, GC suggests that a primary variable can cause a secondary variable if the primary variable’s past data is better than the secondary variable’s past data at predicting the secondary variable’s future (Seth et al., 2015). Luo et al. (2013) use GC to illuminate how the brain exerts top-down control from attention-related regions onto gustatory networks to modulate perception of taste intensity and pleasantness. To accomplish this, subjects were given an MSG stimulus in the scanner and were asked to either remember and rate intensity or pleasantness while another task was interleaved, thereby ensuring that subjects exerted attentional resources. PPIs were constructed using OFC and right AI as seeds, as the former was active during attention over pleasantness, while the latter was active to pleasantness over attention. PPIs revealed stronger connectivity between OFC and aLPFC for attention to pleasantness over intensity. In contrast, for attention to intensity vs. pleasantness, there was greater connectivity between AI and pLPFC. GC was then applied to determine directionality of information flow among these regions. When attending to intensity, subjects exhibited top–down modulation of pLPFC onto AI, as well as bidirectional communication between AI and aLPFC. During attention to pleasantness, top-down modulation of OFC from both aLPFC and pLPFC was observed, as well as indirect communication from pLPFC onto AI and finally to OFC (Luo et al., 2013).

Ge et al. (2012) use a modified version of GC, called componential GC, to both assess interaction effects between variables and compare causal effects across models. Ge et al. (2012) apply componential GC to, like Luo et al. (2013), examine top–down effects of attentional systems when attention is paid to taste intensity vs. pleasantness. To accomplish this, fMRI data were acquired while subjects tasted an MSG stimulus and a tasteless control solution; subjects were instructed to either remember and rate pleasantness or intensity, while trials of unrelated tasks were interleaved. PPIs were constructed using seeds based on *a priori* ROIs obtained from previous literature. PPIs demonstrated greater connectivity between pLPFC and AI, as well as between LPFC and MI, when attention was paid to intensity over pleasantness. Conversely, attention to pleasantness over intensity was associated with greater connectivity between aLPFC and OFC (Ge et al., 2012). GC analyses revealed that, for taste pleasantness, there is bidirectional causal relationship between pLPFC and AI, as well as between AI and aLPFC. In addition, there is top-down modulation of OFC from both pLPFC and aLPFC. Regarding interaction effects, aLPFC’s top–down modulation of OFC during assessment of pleasantness depends on whether OFC is active. GC analysis demonstrated that during attention to intensity, there is a bidirectional causal relationship between OFC and aLPFC and between aLPFC and AI. Last, there is a top–down modulatory effect of pLPFC on AI that also exhibits an interaction effect; indeed, the effect of pLPFC on AI relies on whether AI is active (Ge et al., 2012). Overall, this literature agrees in suggesting that exertion of attention during taste requires modulation of gustatory regions from higher-order regions, such as prefrontal and parietal cortex.

#### Pathology

Two gustatory studies examined patient populations – both related to eating disorders. Frank et al. (2016) examined effective connectivity among food-reward and homeostatic circuitry during sucrose consumption in individuals with anorexia and bulimia nervosa compared to healthy controls. Using Independent Multiple-Sample Greedy Equivalence Search (IMaGES), Frank et al. (2016) found unique patterns in both patient groups; for example, effective connectivity from VS to hypothalamus was directed via ACC, whereas in controls this route excluded ACC. Further, patients exhibited connectivity from substantia nigra to thalamus, with controls exhibiting connectivity in the opposite direction. Sweetness perception itself was predicted via connections to middle OFC; yet this did not differ between patients and controls. Frank et al. (2018) similarly used IMaGES to examine effective connectivity differences between adolescents with anorexia and controls during taste reward conditioning. Notably, in individuals with anorexia, VS drove activation to the hypothalamus, while in controls this pattern was opposite (Frank et al., 2018). Furthermore, in those with anorexia, this pattern of effective connectivity was correlated with prediction error for sucrose in middle and inferior OFC, as well as dorsal AI (Frank et al., 2018). The authors suggest that prediction error signaling from OFC and insula may engage a VS-hypothalamic pathway to potentiate fear and obviate appetitive signals from hypothalamus (Frank et al., 2018).

#### Open Data in Gustation

Of the 15 functional connectivity studies of the gustatory sense, only one of them (i.e., Veldhuizen et al., 2020) included open data. These data are posted on the public repository OpenNeuro^2^.

### Chemesthesis

Chemesthesis research often occurs in the context of other sensory systems, as it is rare for studies to examine the neural correlates of a purely trigeminal stimulus. Of the 12 chemesthesis studies included in this review, all are multisensory; for example, they either include olfactory or gustatory stimuli or study their neural correlates. However, we include chemesthesis research in its own category to reflect the fact that this sense does not fall neatly into either taste or smell. Among all chemesthesis studies are those covering trigeminal networks (*n* = 3), pain (*n* = 4), alcohol (*n* = 3), and other topics (*n* = 2). Also mentioned are studies containing open data.

#### Trigeminal Networks

Of the 12 studies to examine chemesthesis, two used primarily resting-state data. First, Tobia et al. (2016) examined whether olfactory and trigeminal (i.e., chemesthetic) networks are intrinsically organized in the brain; that is, whether they are not simply active in the presence of a relevant task. Seed regions for these networks were obtained from neuroimaging meta-analyses. Significant functional connectivity was observed among olfactory and trigeminal networks at rest. The olfactory network consisted of connectivity between and among hippocampal and parahippocampal regions, thalamus, mPFC, and caudate. The trigeminal network was functionally connected via brainstem, cerebellum, thalamus, caudate, precuneus, and somatosensory cortex. Indeed, these findings demonstrate that both olfactory and trigeminal networks are intrinsically organized and visible in resting-state data.

The second resting-state study of chemesthesis similarly assessed connectivity within olfactory and trigeminal networks, but as a function of aging. Here, Karunanayaka et al. (2017) explicitly follow up on the work of Tobia et al. (2016). A multi-cohort dataset was created with individuals from early to middle adulthood. Resting-state data was collected on each subject, and functional connectivity analyses were performed on seeds within olfactory and trigeminal networks (i.e., those from Tobia et al., 2016). Connectivity between the trigeminal network and ACC, PCC, and parahippocampal gyrus were positively correlated with age. Conversely, functional connectivity between olfactory network and parahippocampal gyrus was negatively associated with age, while connectivity with VS was positively associated with age (Karunanayaka et al., 2017).

#### Pain

Of ten remaining chemesthesis publications, three examined patient populations. One of these, Lee et al. (2021), sought to create a whole-brain functional connectivity “signature” for experimental tonic pain (ETP). They determined to what extent this model could predict various other forms of pain, such as clinical pain and experimental phasic pain (EPP) and whether the underlying neurobiology is similar or different. To accomplish this, tonic pain was induced in subjects by applying capsaicin orally prior to fMRI scanning, with pain ratings measured. Models relating functional connectivity and pain ratings were generated and cross-validated within the capsaicin dataset. These models were validated in a secondary dataset for specificity to pain vs. an aversive taste stimulus that is not painful (i.e., quinine). The best predictive model, termed ToPS, was based on a Brainnetome parcelation of the brain; it used dynamic conditional correlation (DCC) to capture functional connectivity, and principal component regression for model fitting and prediction. A tertiary dataset was used for model validation; the ToPS model exhibited a significant correlation between actual and predicted pain ratings. Further, the model exhibited specificity for pain related to capsaicin, rather than aversive taste (i.e., quinine) or odor (i.e., fermented skate). Finally, the ToPS model could predict pain ratings in patients with clinical back pain, as well as discriminate between patients and controls. Regarding underlying neurobiology, connectivity patterns for ETP and clinical pain were similar in several brain networks but different than EPP; these networks include dorsal attention, somatomotor, and frontoparietal (Lee et al., 2021). Overall, this work yields significant insights, namely that chemesthetic-related pain is transduced differently in the brain than either aversive taste or smell.

While cerebral mechanisms are involved in trigeminal nociception, the brain stem also plays a critical role in this process but arises less frequently in neuroimaging data due to artifacts. To mitigate these issues, Schulte et al. (2016) scanned participants using a protocol optimized for data collection from brain stem; stimulus delivery procedures were the same as those implemented in Hebestreit and May (2017) with the exclusion of drug administration. Data were collected from the entire brain (i.e., inclusive of cerebrum and cerebellum). Both the left spinal trigeminal nucleus (STN) and cuneiform nucleus (CNF) were used as seeds for PPI due to their roles in nociception. The psychological variable of interest was the ammonia vs. air contrast. PPIs revealed that, for this contrast, left STN exhibited greater connectivity with right thalamus, right STN, and right posterior hypothalamus. Regarding CNF, the same psychological condition resulted in greater connectivity with rostral ventromedial medulla.

#### Alcohol

Alcoholic beverages are complex stimuli that engage both gustatory and chemesthetic systems. Ray et al. (2014) recruited subjects with alcohol dependence that were not seeking treatment to participate in an alcohol-cue task during fMRI. PPIs were constructed to determine differences in brain response to alcohol vs. water cues using the VS and caudate as seeds. Decreased functional connectivity was observed between VS and superior lateral occipital cortex, cuneal cortex, and occipital pole for this contrast. Similarly, dorsal striatum (DS) exhibited reduced connectivity in superior lateral occipital cortex, precuneus, fusiform, intracalcarine cortex, and precentral gyrus. While this study aimed to elucidate the neural correlates of alcohol craving, the aforementioned findings have implications for the processing of gustatory vs. chemesthetic stimuli. Filbey et al. (2008) similarly touch on alcohol consumption and seek to examine whether gustatory alcohol cues induce activation of mesocorticolimbic circuitry and whether this correlates with behavioral measures that predict substance abuse. Functional connectivity analyses were performed by taking correlations in time-series among structures in the reward pathway; however, connectivity was not correlated with behavior. A robust connectivity among mesocorticolimbic structures was observed during consumption of alcohol vs. both rest and control. These included between VS and mPFC and OFC, as well as between mPFC and OFC, and between left and right OFC. These findings suggest robust activation of reward regions during consumption of an appetitive chemesthetic stimulus.

Like Filbey et al. (2008), Korucuoglu et al. (2017) elucidate functional connectivity during consumption of alcohol; however, they examine a younger cohort and attempt to tie connectivity to variability in the mu-opioid receptor gene ORPM1. G allele carriers’ receptors exhibit greater affinity for the endogenous ligand, and thus carriers exhibit greater engagement with appetitive stimuli. PPIs were created using seed regions in the right nucleus accumbens and right dorsal caudate; an alcohol vs. water contrast was included as the psychological variable of interest. G allele carriers had greater connectivity between the VS, and MFG, superior frontal gyrus, caudate, middle cingulate, pre/postcentral gyri, and parahippocampus. For the DS, these patterns were observed with hippocampus, thalamus, middle cingulate, precuneus, fusiform, superior parietal lobule, inferior orbital gyrus, inferior occipital gyrus, and middle occipital gyrus (Korucuoglu et al., 2017). These findings differ from those of Filbey et al. (2008) in not finding broad mesocorticolimbic activation, which the authors ascribe to methodological differences. Because of the greater VS-frontal cortex connectivity in G allele carriers to alcohol vs. water, the authors suggest that these individuals exhibit a bias toward reward over cognitive control.

#### Other

The last several chemesthesis studies exclude patient populations and largely use task-based fMRI and PPI analysis. While the latter studies are focused on illuminating the trigeminal system, Rudenga et al. (2010) look to better understand the insula’s role in oral processing. More specifically, Rudenga et al. (2010) examine how the insula responds to whether a stimulus is chemesthetic or gustatory, and whether that stimulus is nutritive or harmful. Subjects underwent fMRI scanning while consuming sucrose, capsaicin, sodium chloride, and quinine solutions. PPIs were constructed using the insula as a seed region and nutritive (sodium chloride and sucrose) vs. harmful (quinine and capsaicin) as the psychological variable of interest. Harmful vs. nutritive conditions did not result in any other brain regions achieving significantly greater connectivity with insula. In contrast, in the nutritive vs. harmful condition, greater connectivity was observed between left insula and VS, ventral pallidum (VP), and hypothalamus (Rudenga et al., 2010). For the same contrast, the right insula exhibited greater connectivity with hypothalamus and bilateral VP.

While evidence for Pavlovian fear conditioning abounds in auditory, somatosensory, and visual domains, the chemosensory domain has been explored less frequently. Because Pavlovian fear conditioning requires the attribution of salience to a previously neutral stimulus, Moessnang et al. (2013) posit that such attribution in the chemosensory domain must be reflected in a common neural currency in salience network. To further explore chemosensory fear conditioning, participants were scanned while one of two odors (rose or vanillin) were paired with an aversive chemesthetic stimulus (CO_2_) (Moessnang et al., 2013). PPIs were constructed using the anterior midcingulate cortex (aMCC) as a seed, as this region has been previously implicated as a convergence site for processing salient stimuli. The psychological variable of interest included whether the conditioned stimulus (CS+) or non-reinforced conditioned stimulus (CS–) was presented. Greater CS+-specific connectivity with aMCC was found in right cerebellum, right sensorimotor cortex, and in both right MI and AI. These findings suggest engagement of salience-related regions during chemosensory Pavlovian fear conditioning, and perhaps convergence of sensory information from trigeminal-olfactory inputs onto these regions.

#### Open Data in Chemesthesis

Of 12 publications to study chemesthesis or use chemesthetic stimuli, only two, Karunanayaka et al. (2017) and Lee et al. (2021) use publicly available data. These data are available on Open Pain^3^ and NITRC^4^, respectively.

### Flavor

Among 103 studies included in this review, a total of 28 are multisensory. That is, apart from those including chemesthetic stimuli or studying chemesthesis, these studies examine a combination of gustation, olfaction, or other senses. First, we elaborate on studies that either examine the neural correlates of flavor (i.e., defined as the combination of gustation and olfaction) or use flavor stimuli. There are eight of these studies, though we only discuss two here to discern between those using flavor stimuli and those studying *neural correlates* of flavor more specifically. These two studies are among several that touch on pleasantness (*n* = 1) and stimulus quality (*n* = 2); the remaining publications (“other,” *n* = 5) are listed in Supplementary Table 2.

#### Pleasantness

Of eight included studies to study flavor explicitly or use stimuli with flavor, most implement a mix of ICA, PPI, or correlation. First, Dalenberg et al. (2017) sought to determine functional networks associated with flavor pleasantness. To accomplish this, two independent datasets were analyzed; in both datasets, participants evaluated flavor pleasantness during fMRI after either drinking common grocery beverages or nutritional supplements. ICA was implemented to determine spatially orthogonal functional networks. A single independent component for common grocery beverage products was correlated with pleasantness; this component contained the ventral emotion network, which consists of ventral PFC, VS, insula, right amygdala, and left parahippocampal gyrus. For the nutritional supplement group, there was no single component associated with flavor pleasantness, though a component containing the ventral emotion network was found (Dalenberg et al., 2017).

#### Quality

Finally, Kudela et al. (2019) use dynamic functional connectivity (dFC), which characterizes the change in functional connectivity through time, to better understand neural representation of flavor. Healthy drinkers underwent fMRI scanning while beer and Gatorade^®^ were delivered. Subjects’ brains were parcellated, using a functional atlas, into seven resting-state networks. dFC for each subject was estimated using a sliding window and bootstrapping procedure; population level dFC was estimated using generalized additive mixed models. Static functional connectivity was also estimated. Greater dFC for beer over Gatorade arises in connections from visual regions to frontoparietal and ventral attention networks (VAN). Gatorade over beer is represented in connectivity within the VAN. A dFC summary metric found significant beer-related association with VS, insula, and OFC, which are regions associated with reward. No differences in static functional connectivity were observed.

#### Open Data in Flavor

Of the studies included in the flavor category, only one, Dalenberg et al. (2017), contains open data in the supplementary information.

### Other Multisensory

Among 20 remaining studies in the multisensory category, these include publications that examine various combinations of olfaction, gustation, and other senses. Of these 20 studies, a total of 17 implement task-based fMRI, one uses PET, and two studies employ resting-state in combination with either task-based fMRI or EEG respectively. In addition to discussing publications with open data, studies of emotion (*n* = 3), olfactory-visual integration (*n* = 4), attention (*n* = 1), and pathology (*n* = 4) are summarized. Eight studies covering other topics are not included here, but are touched on further in Supplementary Table 2.

#### Emotion

Olfactory cues are pivotal in emotion integration; for example, aversive odors can represent “emotional cues” that trigger avoidance. Evidence suggests that odors can trigger similar reactions to subsequent visual cues (e.g., faces). Novak et al. (2015) explore exactly this topic by pairing neutral or fearful faces and aversive odors. “Subthreshold” faces were also created by morphing fearful and neutral faces to investigate less overt effects. Negative odors were also included at various intensities. The fMRI paradigm consisted of odor-visual pairings, and subjects were required to indicate whether a negative emotion was present. Visual, olfactory, and limbic areas were used as ROIs in various DCM models. Visual DCMs indicated bidirectional effective connectivity between extrastriate cortex (EC) and posterior STS, between posterior STS and amygdala, and between amygdala and EC. Driving input from faces entered EC and congruent vs. incongruent stimuli influenced connections from amygdala to posterior STS. For olfactory network, driving input from odors entered PPC, with bidirectional connectivity between PPC and both OFC and amygdala, and between amygdala and OFC (Novak et al., 2015). Congruent vs. incongruent stimuli influenced connections between amygdala and OFC. These findings lend credence to the notion that multisensory regions of convergence, such as amygdala, play a role in odor-vision emotion integration.

Emotions can be encoded in olfactory stimuli, not just from their intensity or aversiveness, but also by their physiological effects. For example, androstadienone or AND, is a pheromone in human sweat that increases the salience of emotional stimuli. To better understand the central effects of AND, Hummer et al. (2017) scanned subjects under AND and a control stimulus, with variously valenced face stimuli presented thereafter. DCMs were created to explore effective connectivity, with ROIs established for amygdala and visual cortex (VC) as well as regions exhibiting effects of AND administration on emotion (i.e., right OFC and PFC). The optimal DCM consisted of driving visual input into VC, with bidirectional connectivity between amygdala and VC and among amygdala, OFC, and PFC (Hummer et al., 2017). Negative valence increased VC-amygdala connectivity; positive images increased this connectivity when a control solution was administered, but not when AND was administered. Notably the lack of an effect from AND potentiated connectivity in OFC and PFC, thus suggesting that AND increases attention to positive stimuli.

#### Olfactory-Visual Integration

Humans integrate sensory information across multiple modalities, with olfactory and visual information being no exception. Ripp et al. (2018) used graph theoretic metrics to better understand networks that exhibit multisensory integration processing (MIP) vs. those that do not, for visual and olfactory stimuli. To accomplish this, subjects were scanned while exposed to unpleasant, neutral, and pleasant pairs of odors and pictures. Connectivity matrices were calculated based on 281 ROIs, and networks were broken down into those processing unimodal stimuli (i.e., olfactory or visual) vs. bimodal stimuli (i.e., both olfactory and visual) (Ripp et al., 2018). The bimodal network exhibited greater correlation between right putamen and right insula, between right precuneus and left supramarginal gyrus, and between left middle occipital gyrus and left IFG compared to the unimodal network. Networks can be further broken down into positive and negative networks (i.e., based on correlations between nodes). Bimodal and unimodal networks can also be compared on their subcomponents to examine MIP more specifically (i.e., where the bimodal olfactory pleasant, visual pleasant is different from the unimodal olfactory pleasant + visual pleasant). Increases in global efficiency and clustering coefficient were observed across all unimodal-bimodal comparisons for both positive and negative networks. Such findings demonstrate that integrated sensory processing networks exhibit more efficient architecture than even the sum of their unimodal parts.

The aforementioned work demonstrates that multisensory integration is complex, but does not explicitly detail what happens when a stimulus in one domain is incongruent with a stimulus in another. Sijben et al. (2018) accomplished exactly this, scanning participants while odor-visual pairs were presented. These stimuli exhibited degrees of healthfulness, and pairs were either incongruent, semi congruent, or congruent. For example, an apple(image)-chocolate(smell) pair is incongruent, but an apple(image)-orange(smell) pair is semi congruent due to matching healthfulness. PPIs were created using ROIs based on both prior literature and significant regions from the main effect of condition. Contrasts were created for every pairwise combination of congruent, semi congruent, and incongruent. Across multiple PPI contrasts, bilateral IFG was connected to a multitude of seeds, including piriform, putamen, left MFG, right STS, and right supramarginal gyrus. Bilateral insula was also connected to multiple seeds, including piriform cortex, right putamen, right STS, and left MFG. These findings suggest a network, consisting of insula and IFG, that respond to congruency levels between stimuli.

As a final work in this domain, Karunanayaka et al. (2015) explored whether learning and memory regions play a role in odor-visual integration, as well as the role of odor intensity on this process. Odor-visual and visual only images were displayed to subjects under fMRI, with odors ranging from fresh air to a weak, medium, strong, and very strong lavender scent. ICA was used to discern functional networks, and unified structural equation modeling (uSEM) was used to determine effective connectivity between the olfactory network and learning and memory-related regions. Two primary olfactory network components were both elicited by ICA, though interestingly, visual only stimuli induced activation in these networks but only when preceded by odor + visual. This effect was dependent on intensity. Further, uSEM found connectivity from olfactory cortex to insula, from insula to OFC and hippocampus, and from hippocampus to olfactory cortex. These regions are critical for associative learning and in memory encoding and retrieval. Overall, these findings demonstrate robust learning of odor + visual pairings, which are intensity dependent and trigger activation in learning and memory structures (Karunanayaka et al., 2015).

#### Attention

Sarinopoulos et al. (2006) evaluate how expectations affect brain networks to influence the experience of aversive taste. During fMRI, subjects were presented with symbols that corresponded to the delivery of tastes at different pleasantness levels. After subjects first learned which visual symbols corresponded to which tastes, some “misleading” symbols were presented (e.g., a mildly aversive symbol paired with highly aversive taste). Functional connectivity analyses were conducted using insula and amygdala as seeds; connectivity was assessed between these regions and both OFC and rostral ACC (rACC) during aversive and misleading conditions. Subjects rated the highly aversive taste more negatively when paired with a matching cue than the same taste when paired with a misleading cue. For the misleading vs. aversive contrast, connectivity was greater between right insula and right OFC and between left insula and rACC (Sarinopoulos et al., 2006). These findings suggest that, under misleading conditions, gustatory regions may be affected by expectancy processing regions to induce a “placebo effect” that dampens the experience of aversive taste.

Distraction, like expectation, may also attenuate taste processing. Furthermore, eating while distracted has been associated with overconsumption and subsequent morbidity. Duif et al. (2020) scanned subjects while they completed both high and low load categorical vision detection tasks, in which high and low sweetness chocolate milk were delivered. After fMRI, subjects watched a documentary, were provided candy, and told to eat until comfortably full. PPIs were created using “taste-related” seed regions (right insula in particular) from the high vs. low sweetness contrast. Psychological variables included all load by sweetness combinations (i.e., low load and low sweetness, low load and high sweetness, etc.). Under high vs. low distraction and high vs. low sweetness, decreased connectivity was found between right insula and right OFC; this suggests a disruption in communication between primary and secondary taste regions in the presence of distraction. Further, reduced insula response during low sweetness consumption promoted subsequent feeding. This suggests that distraction may affect gustatory processing and promote feeding via a network comprising insula and OFC (Duif et al., 2020).

#### Pathology

The first resting-state study by Avery et al. (2018) examined neural correlates of taste reactivity in those with autism (ASD), as these individuals frequently exhibit sensory issues with respect to taste and texture. Both ASD and healthy control individuals were recruited; behavioral assessments of food neophobia and taste reactivity were conducted, along with resting-state fMRI and task-based fMRI with food pictures and gustatory mapping (i.e., sucrose delivery) conducted separately. Seed-based functional connectivity was used with ROIs obtained from the group by taste interaction from the gustatory task. Heightened taste reactivity in ASD was positively correlated with connectivity between bilateral anterior superior temporal sulcus (aSTS) and bilateral mid insula, with a negative correlation observed in controls. This suggests that greater taste reactivity is represented in enhanced taste-related input from insula into aSTS. Avery et al. (2018) argue this reflects a reorganized role of aSTS from processing social stimuli to processing basic sensory information.

A subsequent work to examine patient populations included a cohort with AD. Olfactory impairment is common in neurodegenerative diseases but is characteristic of AD due to the closeness of olfactory and memory structures. Activity in DMN is anticorrelated with memory formation and olfactory processing, and is also impaired in AD. Lu et al. (2019) examined connectivity between olfactory network (ON) and DMN in controls, those with cognitive impairment (MCI; i.e., an intermediary group), and those with AD. Subjects underwent olfactory testing, cognitive evaluation, and an fMRI task with visual and odor-visual stimuli. ICA, volumetric assessments, and effective connectivity analyses (via extended unified structural equation modeling or euSEM) were performed. ICA revealed lowered activation in both ON and DMN, decreasing from controls to MCI and to AD subjects (Lu et al., 2019). Average ON activation across visual and odor-visual conditions demonstrated dysfunctional activity in AD patients compared to MCI and control subjects. Impaired suppression of DMN was observed in AD, and lower hippocampal and olfactory cortex volumes were observed in both AD and MCI, which in turn correlated with measures of smell and memory. Finally, effective connectivity between DMN and ON in MCI was positively correlated with smell measures; this effect was dependent on memory ability Lu et al. (2019). In sum, these findings suggest ON, DMN, and ON-DMN dysfunction in AD and MCI.

Olfactory ability can be affected negatively following stress or aging. Conversely, such conditions may also influence neural processing of olfactory signals emitted by humans via pheromones. In this vein, Maier et al. (2020) investigated impacts of childhood maltreatment (CM) on neural processing of oxytocin and of threat signals present in sweat. Healthy subjects were recruited and assessed for CM. At the beginning of the fMRI session, subjects were administered either an oxytocin or placebo odor, with “stress” sweat, “sports” sweat, and a control stimulus delivered during the scan while variously valenced face stimuli were presented. PPIs were created using *a priori* ROIs including amygdala, hippocampus, fusiform, and OFC, with CM scores representing the psychological variable of interest. Greater CM correlated with greater stress-specific connectivity between right amygdala and left medial OFC, ACC, and hippocampus under placebo (Maier et al., 2020). Further, greater CM correlated with greater effects of oxytocin on stress-specific connectivity between right amygdala and left medial OFC. Indeed, Maier et al. (2020) posit that CM results in poorer frontolimbic regulation in response to chemosensory threat signals.

Like stress, age is associated with aberrant olfactory processing. Martinez et al. (2017) explored age-related olfactory decline and sex differences in a cohort of healthy older adults. Olfactory function was assessed, and participants took part in an odor-visual fMRI task in which odors of varying intensities were presented. Further, euSEM was used to examine sex-specific effects on effective connectivity among *a priori* ROIs. Differences in effective connectivity were observed between sexes. In males, the model consisted of unidirectional connections from olfactory cortex to insula, from insula to dlPFC and from olfactory cortex to hippocampus. In females rather, connectivity was bidirectional from insula to olfactory cortex and unidirectional from dlPFC to insula. Age-related olfactory decline was more pronounced in males than females, perhaps suggesting that these differences in effective connectivity networks promote resilience in females (Martinez et al., 2017).

#### Open Data

Of the 20 multisensory studies, a total of 5 have open or “semi-open” data. For example, Han et al. (2018) and Shanahan et al. (2018) post their respective data on NeuroVault^5^, however, these data include statistical maps rather than their full datasets. Lu et al. (2019) and Maier et al. (2020) will make their data available upon request, while Duif et al. (2020) make their data fully open on Elsevier.

## Discussion

After conducting a thorough review of the literature, we uncovered 103 studies implementing functional connectivity analyses to assess chemosensory-related neural correlates. These publications included both task-based and resting-state data, and examined main themes of gustation, olfaction, chemesthesis, flavor, and integrated multisensory work. Within these categories, we observe numerous subcategories of research topics. These include, among others, aspects of stimulus quality, intensity, and pleasantness. Further, we observe studies of morbidity, such as those related to obesity, Alzheimer’s disease, and anosmia, among others. Finally, many publications examined neural correlates of attention and emotion, as well as studies of multisensory integration (i.e., olfactory-vision).

### Connectivity in Chemosensory Neuroimaging

Among studies examined, we observe several key takeaways. For example, we note conflicting evidence as to whether non-nutritive sweeteners are associated with increased functional connectivity among taste-related brain regions such as FO/AI, or decreased functional connectivity overall (Frank et al., 2008; Van Opstal et al., 2019). We found that unpleasant smells are associated with increased connectivity between prefrontal regions and limbic regions, while unpleasant tastes are associated with increased connectivity between FO and frontal, motor, and reward-related regions (Jabbi et al., 2008; Carlson et al., 2020). Pleasantness of flavor can be attributed to many of these same regions, including insula, prefrontal, and limbic regions (Dalenberg et al., 2017). We note that taste intensity may be influenced by thalamus and insula, while amygdala may act as a CGM (Yeung et al., 2016; Veldhuizen et al., 2020). Pain induced by chemesthetic stimuli may furthermore be associated with activity in dorsal attention, somatomotor, and frontoparietal networks, but the role of brainstem structures should also be considered (Schulte et al., 2016; Lee et al., 2021). We finally note the role of ON-DMN interactions in pathology, as well as the importance of olfactory-visual studies in examining multisensory integration (Ripp et al., 2018; Lu et al., 2019). Indeed, there is a broad array of research in these domains, with few topics untouched. Overall, we note an average sample size of approximately 44 among studies included, which is considered low in the context of task-based fMRI studies (Table 1; Turner et al., 2018). Moreover, we found only 13 of 103 studies (∼12.6%) including open data, (which necessarily hinders study reproducibility), as well as a lack of resting-state and genomics data. We therefore call for more research using open data, comprehensive data types, highly powered neuroimaging datasets, and enhanced methods to optimize the study of chemosensory neural correlates.

### Open Data in Chemosensory Neuroimaging

From studies on other sensory modalities, chemosensory perception is unlikely to be reduced to a single region (e.g., piriform, insula, or postcentral gyrus), but rather interconnected by mesoscale patterns spanning multiple cortical and subcortical systems. To predict on this scale with accuracy, a standardized repository of imaging data is needed (Button et al., 2013). Parcellations of chemosensory areas are already being created with open-access data made available by the HCP (Van Essen et al., 2013). However, understanding how these brain regions interact in a functioning system will take openly published data specific to tasks. Due to the large individual variation or scope of stimuli, several chemosensory tasks have not been studied or have been unable to be modeled by neural signature. For instance, bitterness has known genetic variations leading to large variance in intensity ratings (Prutkin et al., 2000) while several hundred odor qualities exist (Bushdid et al., 2014). Large, pooled samples are needed both to increase the power of models to detect relevant perceptual networks and validate predictive models through internal and external sources (Scheinost et al., 2019). In addition, studies in several chemosensory domains, and olfaction in particular, emphasize clinical over healthy populations, thus excluding analysis on perceptual parts of smelling. However, many of these clinically relevant studies have control arms of healthy subjects. If studies made data publicly available, pooling studies could inform perceptual aspects of a working olfactory system. Studies vary across odorants and concentrations delivered to study subjects, thus opening the possibility of studying odor intensity or quality encoding among large sample pools. Similarly, meta-analysis of spot analysis (such as Activation Likelihood Estimation, or ALE) on olfactory stimulation has helped determine primary regions of activation (Albrecht et al., 2010). ALE has only been possible through the convention of openly reporting XYZ brain coordinates of peak activations; however, such conventions are not in place for connectivity measures. We therefore encourage the use of open data in chemosensory neuroimaging to allow for the exploration of less commonly studied populations, which could mitigate knowledge gaps.

Large, open datasets can also help reduce a known issue of connectivity analysis: inflation of effect sizes (Reddan et al., 2017). Effect size is a unitless description of the strength of a brain-outcome relationship that becomes biased by large amounts of statistical tests often accompanying connectivity analysis approaches (e.g., whole-brain corrections). We note here that the average sample size for a study included in this review is approximately 44, with large variability between studies (Table 1). Indeed, there is utility in sharing data to achieve higher statistical power, as a higher *N* may allow for detection of subtle effects frequently present in neuroimaging (Smith and Nichols, 2018). In addition, shared, open data may be an optimum way to achieve access to larger datasets as it is too costly for one group to obtain. As mentioned, patient-focused studies would not only help with predictive models of unhealthy sensory systems, but may also help understand fully functional ones as their control arms are often healthy. Finally, while shared, large datasets are desirable, there are several challenges inherent to “big data” neuroimaging of which researchers should remain cognizant, including, for example, not only higher sensitivity to true signal but also higher sensitivity to artifact (Smith and Nichols, 2018).

Several large-scale open neuroimaging datasets have emerged over the last several years. These shared datasets have accelerated the progress of neuroscience, increasing collaboration, transparency, and reproducibility (Milham et al., 2018). Datasets are either hosted by data repositories managed through project leads (e.g., UK Biobank; Sudlow et al., 2015) or open repositories which host these datasets as well as user submitted ones (e.g., OpenNeuro). Datasets in either scenario may have different access restrictions. Open neuroimaging datasets may be broad to encompass a population-level sample, or specific to a patient population or a set of behaviors. From the larger open neuroimaging datasets, only one of these datasets (HCP) includes chemosensory measurements (containing an odor identification test). Additionally, several clinically focused open datasets cover etiologies that accompany chemosensory dysfunction (e.g., Philadelphia Neurodevelopmental Cohort and the NKI-Rockland sample) (Nooner et al., 2012; Satterthwaite et al., 2014). Some of these state-of-the-art open datasets have reached their target number of participants, but others are ongoing. Encouraging the inclusion of chemosensory measurements could lead to a better understanding of healthy chemosensory systems and divergent networks in related disease.

Horien et al. (2021) has recently proposed standards for how to work with these larger, open neuroscience datasets. Additionally, they provide some guidance on how to share your own data and what shared data would be useful to aid scientific discovery. In brief, guidance is given on obtaining and managing data, getting to know your data, and communicating results (Horien et al., 2021). As more chemosensory behavioral measures are integrated in larger dataset projects or individual labs share their data in open repositories, researchers wanting to answer specific questions will need to obtain relevant data. Neuroimaging data available or intended to be shared may be raw or processed. Raw data provides the most flexibility, and may come as digital imaging and communication in medicine (DICOM) or neuroimaging informatics technology initiative (NIfTI) images; however, these files are much larger (taking considerable time to download/upload and more resources to host) and require further preprocessing steps (which can be time intensive) prior to analysis. Preprocessed files, such as connectivity matrices or activation maps, can be quickly used by other researchers if shared; however, they may not be appropriate for chemosensory studies (e.g., their parcellations may lack sensory-specific nodes). Images may also be related to structural or diffusion MRI as well as resting-state and/or task fMRI. The researcher must also consider what other data is available or will be shared in an open repository. Other data may include, but not be limited to, behavioral, genomics, angiography, and physiological metrics. To use or share data may require ethical approval [e.g., via institutional review boards (IRB)] as well as getting or setting access approval. Therefore, knowing your data and its source needs to be addressed prior to study proceedings. After study completion, researchers need to communicate their data in a clear, concise way. This allows others to find their data useful and know exactly how that data was used, such that results could be subsequently replicated. Guidelines for reporting neuroimaging methods and results have been made with the Committee on Best Practices in Data Analysis and Sharing (COBIDAS) (Nichols et al., 2017).

### Data-Driven Methods

Of the 103 studies included in this review, the vast majority either use DCM, PPIs, GC, seed-based functional connectivity analyses, or some combination. These tools frequently necessitate the use of *a priori* ROIs that are often based on prior literature or hypotheses regarding how neural systems behave. However, what if prior literature is wrong? For years, cognitive neuroscientists have alluded to a reproducibility crisis, citing scientists’ subconscious (or conscious) preference for “story” over reliability (Huber et al., 2019). What if preconceived hypotheses are incorrect? Rates of confirmed hypotheses in the sciences have been found to range from 70% to a high of 92% in psychiatry and psychology (Fanelli, 2010; Asendorpf et al., 2016). These data suggest that either some scientists write their hypotheses *post hoc*, or that they actively work to confirm them. Because of these potential biases, studies of functional and effective connectivity should seek to use “model free” or data-driven approaches. Such techniques have frequently been used in the predictive modeling literature to form brain-behavior relationships that are less liable to contain the aforementioned forms of bias. For example, one such approach, CPM, has been previously used to generate functional networks that predict personality, waist circumference, and attention (Finn et al., 2015; Rosenberg et al., 2016; Shen et al., 2017; Hsu et al., 2018; Farruggia et al., 2020); it has even been used in a chemosensory capacity to discern individuals with anosmia from those with normosmia (Pellegrino et al., 2021). The utility of such a method is not simply in its capacity to predict behavior based on brain function, but rather its ability to generate predictive features that may yield insight into how the brain truly works. For example, as mentioned previously, Pellegrino et al. (2021) were able to implicate regions beyond olfactory cortex, such as vmPFC and AI, in discriminating between individuals with anosmia and those with normosmia. Indeed, here CPM indicates heretofore novel regions in brain-behavior prediction, suggesting that vmPFC and AI may be involved in the etiology of post-traumatic anosmia. Similar approaches to CPM include other “proprietary” methods like NBS-predict, or machine learning methods such as neural networks, penalized regression, partial least squares regression, and support vector machines and regression (Scheinost et al., 2019; Serin et al., 2021). There are also several “multi-task” methods available to predict more than one behavioral variable at a time; these include, for example, M3T, GGML, MMR, G-SMuRFS, among others (Sui et al., 2020). We recommend considering one or more of these approaches in subsequent data-driven studies of chemosensory-brain relationships. In consonance, we also advise readers to consider best practices in chemosensory neuroimaging before designing study methods (see Veldhuizen, 2022).

### Underutilized Data Types

We note that of all data types included, both genomics (2.91% of studies, Table 1) and resting-state data (26.21% of studies) were relatively underutilized. Additional research using genomics data is imperative, as interindividual differences in chemosensory perception may be mediated by genes, as evidenced in the literature (Newcomb et al., 2012). Functional connectivity studies can therefore be useful in illuminating neural correlates of perceptual differences, as well as in demonstrating how underlying genetics give rise to variable neural architectures. In parallel, resting-state data is imperative in our understanding of how chemosensory networks behave and organize in the absence of perceptual stimuli. Resting-state data is easy to collect, does not require the complex machinery inherent to olfactometers or gustometers, and can be used in combination with perceptual data collected outside of the scanner. Moreover, the act of sniffing, sipping, or chewing commonly present in task-based fMRI paradigms of chemosensory perception induces motion artifacts, which is a pervasive problem in neuroimaging. While resting-state data does not wholly alleviate motion artifacts, it does represent an alternative data type that researchers should consider.

## Conclusion and Limitations

In total, we found 103 studies containing functional connectivity analyses of chemosensory perception. These cover topics of olfaction, gustation, chemesthesis, flavor, and multisensory perception. Among these publications, we noted common themes of research such as multisensory integration, effects of attention and emotion on perception, neural correlates of stimulus, identity, intensity, and pleasantness, and effects of pathologies on chemosensory networks. We note that many of these studies use hypothesis-driven analyses on task-based fMRI data, with a relative lack of genomics literature and open data practices. Therefore, we call for an embracement of big data, open science, and data-driven methods to parallelize chemosensory neuroimaging with other neuroimaging subfields. This review can not only serve as a summary of the state-of-the-art, but also as a motivation to innovate. We note that while we attempt to be comprehensive in our review, we acknowledge common limitations of our search procedure, such as not capturing all relevant keywords or inherent indexing properties that may exclude relevant studies from search databases.

## Author Contributions

MCF, DS, and RP: conceptualization and writing – review and editing. MCF and RP: investigation, formal analysis, visualization, and writing – original draft. DS: supervision and funding acquisition. All authors contributed to the article and approved the submitted version.

## Conflict of Interest

The authors declare that the research was conducted in the absence of any commercial or financial relationships that could be construed as a potential conflict of interest. The handling editor declared a past collaboration with the authors, MCF.

## Publisher’s Note

All claims expressed in this article are solely those of the authors and do not necessarily represent those of their affiliated organizations, or those of the publisher, the editors and the reviewers. Any product that may be evaluated in this article, or claim that may be made by its manufacturer, is not guaranteed or endorsed by the publisher.

## Footnotes

1
https://tinyurl.com/3yb3kdtu

2
https://openneuro.org/

3
https://www.openpain.org/

4
https://www.nitrc.org/

5
https://neurovault.org
