# Supplementary material for: Functional Connectivity of the Chemosenses: A Review
Source: Front Syst Neurosci. 2022 Jun 22;16:865929. doi: 10.3389/fnsys.2022.865929 (PMC9257046; doi:10.3389/fnsys.2022.865929)
Supplement: Supplementary file 1 [file Table_1.docx]

**Table S1.** Full list of studies eligible for inclusion in this review, color coded by category. All titles are included in References.

(Olfaction, Red; Gustation, Orange; Flavor, Green; Chemesthesis, Blue; Multimodal, Purple)

| **Title** | **First Author/ Year** | **Olfactory** | **Gustatory** | **Chemesthesis** | **Flavor** | **Other Senses** |
| --- | --- | --- | --- | --- | --- | --- |
| A Common Anterior Insula Representation of Disgust Observation, Experience and Imagination Shows Divergent Functional Connectivity Pathways | Jabbi et al. (2008) | 0 | 1 | 0 | 0 | 0 |
| Advanced time-series analysis of MEG data as a method to explore olfactory function in healthy controls and Parkinson's disease patients | Boesveldt  et al. (2009) | 1 | 0 | 0 | 0 | 0 |
| A hedonically complex odor mixture produces an attentional capture effect in the brain | Grabenhorst et al. (2011) | 1 | 0 | 0 | 0 | 0 |
| A human chemosignal modulates frontolimbic activity and connectivity in response to emotional stimuli | Hummer et al. (2017) | 1 | 0 | 0 | 0 | 1 |
| Analysis of Mutual Information Content for EEG Responses to Odor Stimulation for Subjects Classified by Occupation | Min et al. (2003) | 1 | 0 | 0 | 0 | 0 |
| A neuroimaging biomarker for sustained experimental and clinical pain | Lee et al. (2021) | 1 | 1 | 1 | 0 | 0 |
| Altered structural and effective connectivity in anorexia and bulimia nervosa in circuits that regulate energy and reward homeostasis | Frank et al. (2016) | 0 | 1 | 0 | 0 | 0 |
| An fMRI Study of the Interactions Between the Attention and the Gustatory Networks | Veldhuizen et al. (2012) | 0 | 1 | 0 | 0 | 0 |
| Association of Brain Reward Learning Response with Harm Avoidance, Weight Gain,  and Hypothalamic Effective Connectivity in Adolescent Anorexia Nervosa | Frank et al. (2018) | 0 | 1 | 0 | 0 | 0 |
| Association of Olfactory Training with Neural Connectivity in Adults with Postviral Olfactory Dysfunction | Jiramongkolchai et al. (2021) | 1 | 0 | 0 | 0 | 0 |
| A study of neural activity and functional connectivity within the olfactory brain network in Parkinson's disease. | Georgiopoulos  et al. (2019) | 1 | 0 | 0 | 0 | 0 |
| Attention to odor modulates thalamocortical connectivity in the human brain | Plailly et al. (2008) | 1 | 0 | 0 | 0 | 0 |
| Attention-dependent modulation of cortical taste circuits revealed by Granger causality with signal-  dependent noise | Luo et al. (2013) | 0 | 1 | 0 | 0 | 0 |
| Attentional modulation of affective versus sensory processing: functional connectivity and a top-down biased activation theory of  selective attention | Grabenhorst et al. (2010) | 0 | 1 | 0 | 0 | 0 |
| Basolateral amygdala response to food cues in the absence of hunger is associated with weight gain susceptibility | Sun et al. (2015) | 0 | 0 | 0 | 1 | 0 |
| Beef assessments using functional magnetic resonance imaging and sensory evaluation | Tapp et al. (2017) | 0 | 0 | 0 | 1 | 0 |
| Behavioral and Neural Changes Induced by a Blended Essential Oil on Human Selective Attention | Liu et al. (2019) | 1 | 0 | 0 | 0 | 0 |
| Brain activity and connectivity changes in response to nutritive natural sugars, non-nutritive natural sugar replacements and artificial sweeteners | Van Opstal et al. (2019) | 0 | 1 | 0 | 0 | 0 |
| Brain Mapping-Based Model of [Delta]9-Tetrahydrocannabinol Effects on Connectivity in the Pain Matrix | Walter et al. (2016) | 1 | 0 | 1 | 0 | 0 |
| Brain mechanisms of expectation associated with insula and amygdala response to aversive taste: Implications for placebo | Sarinopoulos et al. (2006) | 0 | 1 | 0 | 0 | 1 |
| Brain substrates of unhealthy versus healthy food choices: influence of homeostatic status and body mass index | Harding et al. (2018) | 0 | 1 | 0 | 1 | 1 |
| Characterizing functional pathways of the human olfactory system | Zhou et al. (2019) | 1 | 0 | 0 | 0 | 0 |
| Componential Granger causality, and its application to identifying the source and mechanisms of the top-down biased activation that controls attention to affective vs sensory processing | Ge et al. (2012) | 0 | 1 | 0 | 0 | 0 |
| Configural and elemental coding of natural odor mixture components in the human brain | Howard et al. (2014) | 1 | 0 | 0 | 0 | 0 |
| Connectivity of the amygdala, piriform, and orbitofrontal cortex during olfactory stimulation: a functional MRI study | Nigri et al. (2013) | 1 | 0 | 0 | 0 | 0 |
| Converging prefrontal pathways support associative and perceptual features of conditioned stimuli | Howard et al. (2016) | 1 | 0 | 0 | 0 | 0 |
| Default Mode Network Deactivation During Odor-Visual Association | Karunanayaka et al. (2017) | 1 | 0 | 0 | 0 | 1 |
| Different Food Odors Control Brain Connectivity in Impulsive Children | De Celis-Alonso et al. (2019) | 1 | 0 | 0 | 0 | 0 |
| Different patterns of age-related central olfactory decline in men and women as quantified by olfactory fMRI | Martinez et al. (2017) | 1 | 0 | 0 | 0 | 1 |
| Disrupted Olfactory Integration in Schizophrenia: Functional Connectivity Study | Kiparizoska et al. (2017) | 1 | 0 | 0 | 0 | 0 |
| Disruptions of the olfactory and default mode networks in Alzheimer's disease | Lu et al. (2019) | 1 | 0 | 0 | 0 | 1 |
| Effects of chronic peripheral olfactory loss on functional brain networks | Kollndorfer et al. (2015) | 1 | 0 | 0 | 0 | 0 |
| Effects of distraction on taste-related neural processing: a cross-sectional fMRI study | Duif et al. (2020) | 0 | 1 | 0 | 0 | 1 |
| Emotional responses to pleasant and unpleasant olfactory, visual, and auditory stimuli: a positron emission tomography study | Royet et al. (2000) | 1 | 0 | 0 | 0 | 1 |
| Enhanced Olfactory Sensory Perception of Threat in Anxiety: An Event-Related fMRI Study | Krusemark et al. (2012) | 1 | 0 | 0 | 0 | 0 |
| Evidence for an integrated oral sensory module in the human anterior ventral insula | Rudenga et al. (2010) | 0 | 1 | 1 | 0 | 0 |
| Exploring Cortex Connectivity Signal in Sensory Response to Odors | Zhang et al. (2019) | 1 | 0 | 0 | 0 | 0 |
| Exposure to the taste of alcohol elicits activation of the mesocorticolimbic neurocircuitry | Filbey et al. (2008) | 0 | 0 | 1 | 1 | 0 |
| Flavor pleasantness processing in the ventral emotion network | Dalenberg et al. (2017) | 0 | 0 | 0 | 1 | 0 |
| Functional brain networks during picture encoding and recognition in different odor contexts | Reichert et al. (2017) | 1 | 0 | 0 | 0 | 0 |
| Functional Connectivity between the Resting-State Olfactory Network and the Hippocampus in Alzheimer's Disease | Lu et al. (2019) | 1 | 0 | 0 | 0 | 0 |
| Functional Connectome Analyses Reveal the Human Olfactory Network Organization | Arnold et al. (2020) | 1 | 0 | 0 | 0 | 0 |
| Ghrelin Enhances Food Odor Conditioning in Healthy Humans: An fMRI Study | Han et al. (2018) | 1 | 0 | 0 | 0 | 1 |
| Human hippocampal connectivity is stronger in olfaction than other sensory systems | Zhou et al. (2021) | 1 | 0 | 0 | 0 | 1 |
| Human Olfaction without Apparent Olfactory Bulbs | Weiss et al. (2020) | 1 | 0 | 0 | 0 | 0 |
| Identification of an Amygdala-Thalamic Circuit That Acts as a Central Gain Mechanism in Taste Perceptions | Veldhuizen et al. (2020) | 0 | 1 | 0 | 0 | 0 |
| Imaging of odor perception delineates functional disintegration of the limbic circuits in mesial temporal lobe epilepsy | Ciumas et al. (2008) | 1 | 0 | 0 | 0 | 0 |
| Increased functional connectivity within mesocortical networks in open people | Passamonti et al. (2015) | 1 | 0 | 0 | 0 | 0 |
| Individual odor hedonic perception is coded in temporal joint network activity | Ruser et al. (2021) | 1 | 0 | 0 | 0 | 0 |
| Initial evidence that OPRM1 genotype moderates ventral and dorsal striatum functional connectivity during alcohol cues | Ray et al. (2014) | 0 | 0 | 1 | 1 | 0 |
| Intrinsic intranasal chemosensory brain networks shown by resting-state functional MRI | Tobia et al. (2016) | 1 | 0 | 1 | 0 | 0 |
| Investigating the Putative Impact of Odors Purported to Have Beneficial Effects on Sleep: Neural and Perceptual Processes | Ackerley et al. (2020) | 1 | 0 | 0 | 0 | 0 |
| Left Posterior Orbitofrontal Cortex Is Associated with Odor-Induced Autobiographical Memory: An fMRI Study | Watanabe et al. (2018) | 1 | 0 | 0 | 0 | 0 |
| Localization of the primary taste cortex by contrasting passive and attentive conditions | Nakamura et al. (2013) | 0 | 1 | 0 | 0 | 0 |
| Childhood Maltreatment Alters the Neural Processing of Chemosensory Stress Signals | Maier et al. (2020) | 1 | 0 | 0 | 0 | 1 |
| Mere Exposure: Preference Change for Novel Drinks Reflected in Human Ventral Tegmental Area | Ballard et al. (2017) | 0 | 0 | 0 | 1 | 0 |
| Modular structure of functional networks in olfactory memory | Meunier et al. (2014) | 1 | 0 | 0 | 0 | 0 |
| Multisensory integration processing during olfactory-visual stimulation-An fMRI graph theoretical network analysis | Ripp et al. (2018) | 1 | 0 | 0 | 0 | 1 |
| Negative affect and neural response to palatable food intake in bulimia nervosa | Bohon et al. (2012) | 0 | 0 | 0 | 1 | 0 |
| Network organization during probabilistic learning via taste outcomes | Sadler et al. (2020) | 0 | 0 | 0 | 1 | 0 |
| Neural correlates of evaluative compared with passive tasting | Bender et al. (2009) | 0 | 1 | 0 | 0 | 0 |
| Neural correlates of taste reactivity in autism spectrum disorder | Avery et al. (2018) | 0 | 1 | 0 | 0 | 1 |
| Neural correlates of working memory's suppression of aversive olfactory distraction effects | Weigard et al. (2021) | 1 | 0 | 0 | 0 | 0 |
| Neural response to alcohol taste cues in youth: effects of the OPRM1 gene | Korucuoglu et al. (2017) | 0 | 0 | 1 | 1 | 0 |
| Normal Olfactory Functional Connectivity Despite Lifelong Absence of Olfactory Experiences | Peter et al. (2021) | 1 | 0 | 0 | 0 | 0 |
| Odor-evoked category reactivation in human ventromedial prefrontal cortex during sleep promotes memory consolidation | Shanahan et al. (2018) | 1 | 0 | 0 | 0 | 1 |
| Odor identity can be extracted from the reciprocal connectivity between olfactory bulb and piriform cortex in humans | Iravani et al. (2021) | 1 | 0 | 0 | 0 | 0 |
| Olfactory connectivity mediates sleep-dependent food choices in humans | Bhutani et al. (2019) | 1 | 0 | 0 | 0 | 0 |
| Olfactory Dysfunction Mediates Adiposity in Cognitive Impairment of Type 2 Diabetes: Insights from Clinical and Functional Neuroimaging Studies | Zhang et al. (2019) | 1 | 0 | 0 | 0 | 0 |
| Olfactory fMRI: Implications of Stimulation Length and Repetition Time | Georgiopoulos et al. (2018) | 1 | 0 | 0 | 0 | 0 |
| Olfactory Network Differences in Master Sommeliers: Connectivity Analysis Using Granger Causality and Graph Theoretical Approach | Sreenivasan et al. (2017) | 1 | 0 | 0 | 0 | 1 |
| Olfactory performance and resting state functional connectivity in non-demented drug naïve patients with Parkinson's disease | Sunwoo et al. (2015) | 1 | 0 | 0 | 0 | 0 |
| Olfactory training induces changes in regional functional connectivity in patients with long-term smell loss | Kollndorfer et al. (2015) | 1 | 0 | 1 | 0 | 0 |
| Olfactory-visual integration facilitates perception of subthreshold negative emotion | Novak et al. (2015) | 1 | 0 | 0 | 0 | 1 |
| Opposing roles for amygdala and vmPFC in the return of appetitive conditioned responses in humans | Ebrahimi et al. (2019) | 0 | 0 | 0 | 1 | 0 |
| Patterns of olfactory functional networks in Parkinson's disease dementia and Alzheimer's dementia | Lee et al. (2020) | 1 | 0 | 0 | 0 | 0 |
| Physiological brainstem mechanisms of trigeminal nociception: An fMRI study at 3T | Schulte et al. (2016) | 1 | 0 | 1 | 0 | 1 |
| Post-traumatic olfactory loss and brain response beyond olfactory cortex | Pellegrino et al. (2021) | 1 | 0 | 0 | 0 | 0 |
| Rapidly acquired multisensory association in the olfactory cortex | Karunanayaka et al. (2015) | 1 | 0 | 0 | 0 | 1 |
| Recovery of Olfactory Function Induces Neuroplasticity Effects in Patients with Smell Loss | Kollndorfer et al. (2014) | 1 | 0 | 0 | 0 | 0 |
| Rivalry of homeostatic and sensory-evoked emotions: Dehydration attenuates olfactory disgust and its neural correlates | Meier et al. (2015) | 1 | 0 | 0 | 0 | 0 |
| Satiation attenuates BOLD activity in brain regions involved in reward and increases activity in dorsolateral prefrontal cortex: an fMRI study in healthy volunteers | Thomas et al. (2015) | 0 | 0 | 0 | 1 | 1 |
| Semantic Congruence Alters Functional Connectivity during Olfactory-Visual Perception | Sijben et al. (2018) | 1 | 0 | 0 | 0 | 1 |
| Semiparametric Estimation of Task-Based Dynamic Functional Connectivity on the Population Level | Kudela et al. (2019) | 0 | 0 | 0 | 1 | 0 |
| Severe hyposmia and aberrant functional connectivity in cognitively normal Parkinson's disease | Yoneyama et al. (2018) | 1 | 0 | 0 | 0 | 0 |
| Severity of olfactory deficits is reflected in functional brain networks-An fMRI study | Reichert et al. (2018) | 1 | 0 | 0 | 0 | 0 |
| Sucrose activates human taste pathways differently from artificial sweetener | Frank et al. (2008) | 0 | 1 | 0 | 0 | 0 |
| Superadditive opercular activation to food flavor is mediated by enhanced temporal and limbic coupling | Seubert et al. (2015) | 1 | 1 | 0 | 1 | 0 |
| Sustained effects of pleasant and unpleasant smells on resting state brain activity | Carlson et al. (2020) | 1 | 0 | 0 | 0 | 0 |
| Taste intensity modulates effective connectivity from the insular cortex to the thalamus in humans | Yeung et al. (2016) | 0 | 1 | 0 | 0 | 0 |
| Taste laterality studied by means of umami and salt stimuli: an fMRI study | Iannilli et al. (2012) | 0 | 1 | 0 | 0 | 0 |
| The additive effect of late-life depression and olfactory dysfunction on the risk of dementia was mediated by hypersynchronization of the hippocampus/fusiform gyrus | Chen et al. (2021) | 1 | 0 | 0 | 0 | 0 |
| The Anterior Insular Cortex Represents Breaches of Taste Identity Expectation | Veldhuizen et al. (2011) | 0 | 1 | 0 | 0 | 0 |
| The scent of salience -- Is there olfactory-trigeminal conditioning in humans? | Moessnang et al. (2013) | 1 | 0 | 1 | 0 | 0 |
| Topiramate modulates trigeminal pain processing in thalamo-cortical networks in humans after single dose administration | Hebestreit et al. (2017) | 1 | 0 | 1 | 0 | 0 |
| True and False Recognition Memories of Odors Induce Distinct Neural Signatures | Royet et al. (2011) | 1 | 0 | 0 | 0 | 0 |
| Ultra-slow mechanical stimulation of olfactory epithelium modulates consciousness by slowing cerebral rhythms in humans | Piarulli et al. (2018) | 1 | 0 | 0 | 0 | 0 |
| When the sense of smell meets emotion: anxiety-state-dependent olfactory processing and neural circuitry adaptation | Krusemark et al. (2013) | 1 | 0 | 0 | 0 | 0 |
| When to collect resting-state data: The influence of odor on post-task resting-state connectivity | Cecchetto et al. (2019) | 1 | 0 | 0 | 0 | 0 |
| Age-related resting-state functional connectivity in the olfactory and trigeminal networks | Karunanayaka et al. (2017) | 1 | 0 | 1 | 0 | 0 |
| Alterations in striato-thalamo-pallidal intrinsic functional connectivity as a prodrome of Parkinson's disease | Dayan et al. (2017) | 1 | 0 | 0 | 0 | 0 |
| Alterations in the limbic/paralimbic cortices of Parkinson's disease patients with hyposmia under resting-state functional MRI by regional homogeneity and functional connectivity analysis | Su et al. (2015) | 1 | 0 | 0 | 0 | 0 |
| Altered Functional Brain Networks in Patients with Traumatic Anosmia: Resting-State Functional MRI Based on Graph Theoretical Analysis | Park et al. (2019) | 1 | 0 | 0 | 0 | 0 |
