# Supplementary material for: Functional Connectivity of the Chemosenses: A Review
Source: Front Syst Neurosci. 2022 Jun 22;16:865929. doi: 10.3389/fnsys.2022.865929 (PMC9257046; doi:10.3389/fnsys.2022.865929)
Supplement: Supplementary file 2 [file Table_2.docx]

**Table S2.** Full list of all 103 studies eligible for inclusion in this review and their respective summary data.

| **Topic** | **Author(s)** | **Subtopic** | **Patient Group** | **Behavioral** | **Physiological** | **Genomic** | **Imaging Modality** | **Analytic Method** | **Open Data** |
| --- | --- | --- | --- | --- | --- | --- | --- | --- | --- |
| **Olfaction** | Ackerley et al. (2020) | Other | Ｘ | ✓ | Ｘ | Ｘ | task fMRI | Correlation matrix | Ｘ |
|  | Arnold et al. (2020) | Anatomy | Ｘ | Ｘ | Ｘ | Ｘ | rs-fMRI | Seed-based, Parcellation | ✓ |
|  | Bhutani et al. (2019) | Other | Ｘ | ✓ | ✓ | Ｘ | task fMRI | PPI | Ｘ |
|  | Boesveldt et al. (2009) | Pathology | ✓ | ✓ | Ｘ | Ｘ | MEG | Synchronization Likelihood | Ｘ |
|  | Carlson et al. (2020) | Pleasantness | Ｘ | ✓ | ✓ | Ｘ | rs-fMRI | Correlation matrix | ✓ |
|  | Cecchetto et al. (2019) | Other | Ｘ | ✓ | Ｘ | Ｘ | task fMRI, rs-fMRI | Seed-based | Ｘ |
|  | Chen et al. (2021) | Pathology | ✓ | ✓ | Ｘ | Ｘ | rs-fMRI | ReHo | Ｘ |
|  | Ciumas et al. (2008) | Pathology | ✓ | ✓ | ✓ | Ｘ | PET | Covariance of rCBF | Ｘ |
|  | Dayan et al. (2017) | Other | ✓ | ✓ | Ｘ | Ｘ | rs-fMRI | Seed-based | ✓ |
|  | De Celis-Alonso et al. (2019) | Emotion | ✓ | ✓ | Ｘ | Ｘ | task fMRI | Correlation matrix | Ｘ |
|  | Georgiopoulos et al. (2018) | Anatomy | Ｘ | ✓ | Ｘ | Ｘ | task fMRI | ICA | Ｘ |
|  | Georgiopoulos et al. (2019) | Pathology | ✓ | ✓ | Ｘ | Ｘ | task fMRI, rs-fMRI | ICA, Seed-based | Ｘ |
|  | Grabenhorst et al. (2011) | Attention | Ｘ | ✓ | Ｘ | Ｘ | task fMRI | PPI | Ｘ |
|  | Howard et al. (2014) | Other | Ｘ | ✓ | ✓ | Ｘ | task fMRI | PPI | Ｘ |
|  | Howard et al. (2016) | Other | Ｘ | ✓ | Ｘ | Ｘ | task fMRI | PPI | Ｘ |
|  | Iravani et al. (2021) | Anatomy | Ｘ | ✓ | Ｘ | Ｘ | EEG | Cross Spectrogram, Granger Causality | ✓ |
|  | Jiramongkolchai et al. (2021) | Pathology | ✓ | ✓ | Ｘ | Ｘ | rs-fMRI | Seed-based | Ｘ |
|  | Kiparizoska et al. (2017) | Pathology | ✓ | Ｘ | Ｘ | Ｘ | rs-fMRI | Seed-based | ✓ |
|  | Kollndorfer et al. (2014) | Pathology | ✓ | ✓ | Ｘ | Ｘ | task fMRI | Seed-based | Ｘ |
|  | Kollndorfer et al. (2015) | Pathology | ✓ | ✓ | Ｘ | Ｘ | task fMRI | ICA, Correlation matrix | Ｘ |
|  | Krusemark et al. (2012) | Emotion | Ｘ | ✓ | Ｘ | Ｘ | task fMRI | PPI | Ｘ |
|  | Krusemark et al. (2013) | Emotion | Ｘ | ✓ | Ｘ | Ｘ | task fMRI | PPI, DCM | Ｘ |
|  | Lee et al. (2020) | Pathology | ✓ | ✓ | Ｘ | Ｘ | rs-fMRI | Seed-based | Ｘ |
|  | Liu et al. (2019) | Attention | Ｘ | Ｘ | ✓ | Ｘ | EEG | EEG Synch., Phase Locking Value (PLV) | Ｘ |
|  | Lu et al. (2019) | Pathology | ✓ | ✓ | Ｘ | Ｘ | rs-fMRI | Seed-based | ✓ |
|  | Meier et al. (2015) | Emotion | Ｘ | ✓ | ✓ | Ｘ | rs-fMRI | PPI | Ｘ |
|  | Meunier et al. (2014) | Other | Ｘ | ✓ | Ｘ | Ｘ | task fMRI, rs-fMRI | Correlation matrix | Ｘ |
|  | Min et al. (2003) | Other | Ｘ | ✓ | Ｘ | Ｘ | EEG | A-CMI | Ｘ |
|  | Nigri et al. (2013) | Anatomy | Ｘ | ✓ | Ｘ | Ｘ | task fMRI | PPI | Ｘ |
|  | Park et al. (2019) | Pathology | ✓ | Ｘ | Ｘ | Ｘ | rs-fMRI | Correlation matrix | Ｘ |
|  | Passamonti et al. (2015) | Pathology | Ｘ | ✓ | Ｘ | Ｘ | task fMRI, rs-fMRI | PPI, Seed-based | Ｘ |
|  | Pellegrino et al. (2021) | Pathology | ✓ | ✓ | Ｘ | Ｘ | task fMRI | CPM | Ｘ |
|  | Peter et al. (2021) | Pathology | ✓ | ✓ | Ｘ | Ｘ | rs-fMRI | ReHo, Voxel-mirrored Homotopic Connectivity |  |
|  | Piarulli et al. (2018) | Other | Ｘ | ✓ | ✓ | Ｘ | EEG | Granger Causality, CFI | Ｘ |
|  | Plailly et al. (2008) | Attention | Ｘ | ✓ | ✓ | Ｘ | task fMRI | DCM | Ｘ |
|  | Reichert et al. (2017) | Other | Ｘ | ✓ | Ｘ | Ｘ | task fMRI | ICA | Ｘ |
|  | Reichert et al. (2018) | Pathology | ✓ | ✓ | Ｘ | Ｘ | task fMRI | ICA | Ｘ |
|  | Royet et al. (2011) | Other | Ｘ | ✓ | Ｘ | Ｘ | task fMRI | CVA | Ｘ |
|  | Ruser et al. (2021) | Pleasantness | Ｘ | ✓ | ✓ | Ｘ | task fMRI | Correlation matrix | Ｘ |
|  | Su et al. (2015) | Pathology | ✓ | ✓ | Ｘ | Ｘ | rs-fMRI | Seed-based | Ｘ |
|  | Sunwoo et al. (2015) | Pathology | ✓ | ✓ | Ｘ | Ｘ | rs-fMRI | Seed-based, ReHo | Ｘ |
|  | Watanabe et al. (2018) | Other | Ｘ | ✓ | ✓ | Ｘ | task fMRI | PPI | Ｘ |
|  | Weigard et al. (2021) | Attention | Ｘ | ✓ | ✓ | Ｘ | task fMRI | PPI | Ｘ |
|  | Weiss et al. (2020) | Anatomy | ✓ | Ｘ | Ｘ | Ｘ | task fMRI | PPI | ✓ |
|  | Yoneyama et al. (2018) | Pathology | ✓ | ✓ | Ｘ | Ｘ | rs-fMRI | Seed-based, ICA | Ｘ |
|  | Zhang et al. (2019) | Anatomy | Ｘ | Ｘ | ✓ | Ｘ | EEG | Magnitude Squared Coherence Estimation | Ｘ |
|  | Zhang et al. (2019) | Other | ✓ | ✓ | ✓ | Ｘ | rs-fMRI | Seed-based | Ｘ |
|  | Zhou et al. (2019) | Anatomy | Ｘ | Ｘ | Ｘ | Ｘ | rs-fMRI | Correlation matrix, K-means | ✓ |
| **Gustation** | Bender et al. (2009) | Pleasantness | Ｘ | ✓ | Ｘ | Ｘ | task fMRI | PPI | Ｘ |
|  | Frank et al. (2008) | Sweeteners | Ｘ | ✓ | Ｘ | Ｘ | task fMRI | Correlation matrix | Ｘ |
|  | Frank et al. (2016) | Pathology | ✓ | ✓ | Ｘ | Ｘ | task fMRI | IMaGES | Ｘ |
|  | Frank et al. (2018) | Pathology | ✓ | ✓ | ✓ | Ｘ | task fMRI | IMaGES; Linear Nongaussian Orientation | Ｘ |
|  | Ge et al. (2012) | Attention | Ｘ | ✓ | Ｘ | Ｘ | task fMRI | Componential Granger Causality | Ｘ |
|  | Grabenhorst et al. (2010) | Pleasantness | Ｘ | Ｘ | Ｘ | Ｘ | task fMRI | PPI | Ｘ |
|  | Iannilli et al. (2012) | Anatomy | Ｘ | ✓ | Ｘ | Ｘ | task fMRI | DCM | Ｘ |
|  | Jabbi et al. (2008) | Pleasantness | Ｘ | ✓ | Ｘ | Ｘ | task fMRI | PPI | Ｘ |
|  | Luo et al. (2013) | Attention | Ｘ | Ｘ | Ｘ | Ｘ | task fMRI | PPI; Granger Causality | Ｘ |
|  | Nakamura et al. (2013) | Anatomy | Ｘ | ✓ | Ｘ | Ｘ | task fMRI | DCM | Ｘ |
|  | Van Opstal et al. (2019) | Sweeteners | Ｘ | Ｘ | Ｘ | Ｘ | rs-fMRI | ECM | Ｘ |
|  | Veldhuizen et al. (2011) | Attention | Ｘ | ✓ | Ｘ | Ｘ | task fMRI | PPI, DCM | Ｘ |
|  | Veldhuizen et al. (2012) | Attention | Ｘ | Ｘ | Ｘ | Ｘ | task fMRI | PPI, DCM | Ｘ |
|  | Veldhuizen et al. (2020) | Taste Intensity | Ｘ | ✓ | Ｘ | Ｘ | task fMRI | PPI, DCM | ✓ |
|  | Yeung et al. (2016) | Taste Intensity | Ｘ | ✓ | Ｘ | Ｘ | task fMRI | DCM | Ｘ |
| **Chemesthesis** | Filbey et al. (2008) | Alcohol | Ｘ | ✓ | Ｘ | Ｘ | task fMRI | Correlation matrix | Ｘ |
|  | Hebestreit et al. (2017) | Pain | Ｘ | ✓ | Ｘ | Ｘ | task fMRI | PPI | Ｘ |
|  | Karunanayaka et al. (2017) | Trigeminal Networks | Ｘ | Ｘ | Ｘ | Ｘ | rs-fMRI | Seed-based | ✓ |
|  | Kollndorfer et al. (2015) | Trigeminal Networks | ✓ | ✓ | Ｘ | Ｘ | task fMRI | ICA, Seed-based | Ｘ |
|  | Korucuoglu et al. (2017) | Alcohol | Ｘ | ✓ | ✓ | ✓ | task fMRI | PPI | Ｘ |
|  | Lee et al. (2021) | Pain | ✓ | Ｘ | ✓ | Ｘ | task fMRI | Regression, Whole Brain FC | ✓ |
|  | Moessnang et al. (2013) | Other | Ｘ | ✓ | ✓ | Ｘ | task fMRI | PPI | Ｘ |
|  | Ray et al. (2014) | Alcohol | ✓ | ✓ | Ｘ | ✓ | task fMRI | PPI | Ｘ |
|  | Rudenga et al. (2010) | Other | Ｘ | ✓ | Ｘ | Ｘ | task fMRI | PPI | Ｘ |
|  | Schulte et al. (2016) | Pain | Ｘ | ✓ | ✓ | Ｘ | task fMRI | PPI | Ｘ |
|  | Tobia et al. (2016) | Trigeminal Networks | Ｘ | Ｘ | Ｘ | Ｘ | rs-fMRI | Seed-based | Ｘ |
|  | Walter et al. (2016) | Pain | Ｘ | ✓ | Ｘ | Ｘ | task fMRI | PPI, DCM | Ｘ |
| **Flavor** | Ballard et al. (2017) | Other | Ｘ | ✓ | Ｘ | Ｘ | task fMRI | PPI | Ｘ |
|  | Bohon et al. (2012) | Other | ✓ | ✓ | Ｘ | Ｘ | task fMRI | PPI | Ｘ |
|  | Dalenberg et al. (2017) | Pleasantness | Ｘ | ✓ | Ｘ | Ｘ | task fMRI | ICA | ✓ |
|  | Ebrahimi et al. (2019) | Other | Ｘ | ✓ | ✓ | Ｘ | task fMRI | PPI | Ｘ |
|  | Kudela et al. (2019) | Quality | Ｘ | Ｘ | Ｘ | Ｘ | task fMRI | Correlation matrix, dFC | Ｘ |
|  | Sadler et al. (2020) | Quality | Ｘ | ✓ | Ｘ | Ｘ | task fMRI | Correlation matrix | Ｘ |
|  | Sun et al. (2015) | Other | Ｘ | Ｘ | ✓ | ✓ | task fMRI, rs-fMRI | DCM | Ｘ |
|  | Tapp et al. (2017) | Other | Ｘ | ✓ | Ｘ | Ｘ | rs-fMRI | Seed-based | Ｘ |
| **Other Multisensory** | Avery et al. (2018) | Pathology | ✓ | ✓ | Ｘ | Ｘ | rs-fMRI | Seed-based | Ｘ |
|  | Duif et al. (2020) | Attention | Ｘ | ✓ | ✓ | Ｘ | task fMRI | PPI | ✓ |
|  | Han et al. (2018) | Other | Ｘ | ✓ | ✓ | Ｘ | task fMRI | PPI | Ｘ |
|  | Harding et al. (2018) | Other | Ｘ | Ｘ | ✓ | Ｘ | task fMRI | PPI | Ｘ |
|  | Hummer et al. (2017) | Emotion | Ｘ | ✓ | Ｘ | Ｘ | task fMRI | DCM | Ｘ |
|  | Karunanayaka et al. (2015) | O-V Integration | Ｘ | ✓ | Ｘ | Ｘ | task fMRI | ICA, euSEM | Ｘ |
|  | Karunanayaka et al. (2017) | O-V Integration | Ｘ | ✓ | Ｘ | Ｘ | task fMRI | euSEM, PPI | Ｘ |
|  | Lu et al. (2019) | Pathology | ✓ | ✓ | Ｘ | Ｘ | task fMRI | euSEM | Ｘ |
|  | Maier et al. (2020) | Pathology | Ｘ | ✓ | ✓ | Ｘ | task fMRI | PPI | Ｘ |
|  | Martinez et al. (2017) | Pathology | Ｘ | ✓ | Ｘ | Ｘ | task fMRI | euSEM | Ｘ |
|  | Novak et al. (2015) | Emotion | Ｘ | ✓ | ✓ | Ｘ | task fMRI | DCM | Ｘ |
|  | Ripp et al. (2018) | O-V Integration | Ｘ | ✓ | Ｘ | Ｘ | task fMRI | Correlation matrix | Ｘ |
|  | Royet et al. (2000) | Other | Ｘ | ✓ | Ｘ | Ｘ | PET | PCA | Ｘ |
|  | Sarinopoulos et al. (2006) | Emotion | Ｘ | ✓ | Ｘ | Ｘ | task fMRI | Seed-based | Ｘ |
|  | Seubert et al. (2015) | Other | Ｘ | ✓ | Ｘ | Ｘ | task fMRI | PPI | Ｘ |
|  | Shanahan et al. (2018) | Other | Ｘ | ✓ | ✓ | Ｘ | task fMRI | PPI | Ｘ |
|  | Sijben et al. (2018) | O-V Integration | Ｘ | ✓ | ✓ | Ｘ | task fMRI | PPI | Ｘ |
|  | Sreenivasan et al. (2017) | Other | Ｘ | Ｘ | Ｘ | Ｘ | task fMRI | Granger Causality, MVAR | Ｘ |
|  | Thomas et al. (2015) | Other | Ｘ | ✓ | Ｘ | Ｘ | task fMRI | PPI | Ｘ |
|  | Zhou et al. (2021) | Other | ✓ | Ｘ | Ｘ | Ｘ | rs-fMRI, EEG | Seed-based, dwPLI | Ｘ |
